# Phylogenomics of *Psammodynastes* and *Buhoma* (Elapoidea: Serpentes), with the description of a new Asian snake family

**DOI:** 10.1038/s41598-024-60215-2

**Published:** 2024-04-25

**Authors:** Sunandan Das, Eli Greenbaum, Jonathan Brecko, Olivier S. G. Pauwels, Sara Ruane, Stacy Pirro, Juha Merilä

**Affiliations:** 1 Ecological Genetics Research Unit, Organismal and Evolutionary Biology Research Programme, Faculty of Biological and Environmental Sciences, University of Helsinki, 00014 Helsinki, Finland; 2 Department of Biological Sciences, University of Texas at El Paso, 500 W. University Avenue, El Paso, TX 79968 USA; 3 Royal Belgian Institute of Natural Sciences, Rue Vautier 29, 1000 Brussels, Belgium; 4 Royal Museum for Central Africa, Tervuren, Belgium; 5 Life Sciences Section, Negaunee Integrative Research Center, Field Museum, Chicago, IL USA; 6 Iridian Genomes Inc., Bethesda, MD 20817 USA; 7 Area of Ecology and Biodiversity, School of Biological Sciences, Kadoorie Biological Sciences Building, The University of Hong Kong, Pokfulam Road, Hong Kong, SAR China

**Keywords:** Evolution, Zoology

## Abstract

Asian mock vipers of the genus *Psammodynastes* and African forest snakes of the genus *Buhoma* are two genera belonging to the snake superfamily Elapoidea. The phylogenetic placements of *Psammodynastes* and *Buhoma* within Elapoidea has been extremely unstable which has resulted in their uncertain and debated taxonomy. We used ultraconserved elements and traditional nuclear and mitochondrial markers to infer the phylogenetic relationships of these two genera with other elapoids. *Psammodynastes*, for which a reference genome has been sequenced, were found, with strong branch support, to be a relatively early diverging split within Elapoidea that is sister to a clade consisting of Elapidae, Micrelapidae and Lamprophiidae. Hence, we allocate *Psammodynastes* to its own family, Psammodynastidae *new family*. However, the phylogenetic position of *Buhoma* could not be resolved with a high degree of confidence. Attempts to identify the possible sources of conflict in the rapid radiation of elapoid snakes suggest that both hybridisation/introgression during the rapid diversification, including possible ghost introgression, as well as incomplete lineage sorting likely have had a confounding role. The usual practice of combining mitochondrial loci with nuclear genomic data appears to mislead phylogeny reconstructions in rapid radiation scenarios, especially in the absence of genome scale data.

## Introduction

The snake superfamily Elapoidea, with over 700 species, constitutes more than one-fifth of global snake diversity. The superfamily includes two of the major medically important venomous snake clades, namely the cosmopolitan Elapidae and the Afro-Middle Eastern Atractaspidinae (Lamprophiidae), and a diverse radiation of other snakes (namely, Cyclocoridae, Micrelapidae, and subfamilies of Lamprophiidae) spread over Africa, Madagascar, Europe, and Asia. The phylogeny of this major group of snakes was highly debated until recently, with researchers producing strikingly conflicting, poorly supported topologies at deeper levels (e.g.,^1–6^). The recalcitrance of the elapoid phylogeny has been attributed to an ancient, rapid radiation^1^. Recently, Das et al.^7^ used over 4500 ultraconserved elements (UCE) loci and both multispecies coalescent and concatenation-based phylogenomic methods to resolve the backbone of the elapoid phylogeny.

However, the relationships between the major (i.e., those treated at subfamily and family ranks) subclades within Elapoidea have not been the only issue with the phylogenetic systematics of this superfamily. The phylogenetic positions of *Buhoma*, *Micrelaps* and *Psammodynastes* could not be consistently resolved with a limited number of Sanger sequenced loci (e.g.,^1,3,4^). While studies agree^1,3,4,6,11^ that these genera belong to Elapoidea, they have not been assigned to any specific subfamily or family as their positioning within the elapoid phylogeny is unstable across different works. Das et al*.*^7^ were able to infer the phylogenetic placement of *Micrelaps* with a high degree of statistical branch support with UCE data. However, the sampling of Das et al*.*^7^ did not include *Buhoma* and *Psammodynastes* and hence, the phylogenetic positioning of these two genera remains enigmatic. Thorough anatomical comparisons of these two genera with other major elapoid subclades have not been done either (Das et al.^7^ only reported that cranial features do not support the assignment of *Psammodynastes* to Pseudaspidinae).

The Mock Vipers of the genus *Psammodynastes* have an extensive range in south and south-east Asia, including the Indonesian archipelago^8,9^. The genus includes two species—*P*. *pictus* and *P*. *pulverulentus*. These rear-fanged snakes have historically been assigned to the catch-all family Colubridae^10^. The phylogenetic placement of *Psammodynastes* has been contentious. Within Elapoidea, *Psammodynastes* has been placed—I. as sister to Lamprophiidae^11^, II. sister to *Buhoma*, with this cluster not being closely related to Pseudaspidinae^1^, III. basal split to a polytomous clade consisting of Psammophiinae, Prosymninae, Pseudaspidinae and *Buhoma*^2^, IV. sister to *Buhoma*, with this cluster sharing a common ancestor with Pseudaspidinae^3,5^, V. nested within Psammophiinae^4^, and VI. sister to Pseudaspidinae^6^.

The phylogenetic position of the African forest snakes, *Buhoma* spp., has also been highly unstable. Points II, III and IV on the problematic phylogenetic placements of *Psammodynastes* are also pertinent to *Buhoma*. Apart from those, *Buhoma* has also been found to be—I. sister to (*Psammodynastes*, Lamprophiidae)^11^, II. sister to Elapidae^6^, and as III. polyphyletic in the phylogeny of Figueroa et al.^4^, with *B. procterae* being sister to Prosymninae and *B. depressiceps* as sister to Elapidae.

The aim of this study is to resolve the phylogenetic relationships of the genera *Psammodynastes* and *Buhoma* within the Elapoidea and to make appropriate taxonomic and nomenclatural decisions regarding these two genera. We used ultraconserved elements extracted from published and newly generated reference genomes and from a published target capture sequencing dataset to infer the phylogenetic interrelationships of *Psammodynastes* and *Buhoma*. For comparative purposes, we also utilized legacy nuclear and mitochondrial markers for phylogenetic inference. To look for potential apomorphies and/or diagnostic character states, we studied and compared micro-computed tomographic scans of crania of *Psammodynastes*, *Buhoma* and members of every elapoid family/subfamily level clade. We also investigated the sources of conflict in different datasets.

## Results

### Phylogenomics

The 50, 75 and 95% complete UCE datasets consisted of 4514 (~ 5.3 M bp), 3993 (~ 4.7 M bp) and 2467 (~ 2.9 M bp) loci respectively. The concatenated Sanger loci dataset was 7439 bp long (*BDNF* 669 bp, *C-MOS* 735 bp, *RAG1* 1011 bp, *RAG2* 912 bp, *12S* 979 bp, *16S* 1342 bp, *CYTB* 1098 bp and *ND4* 693 bp).

**Figure 1:**
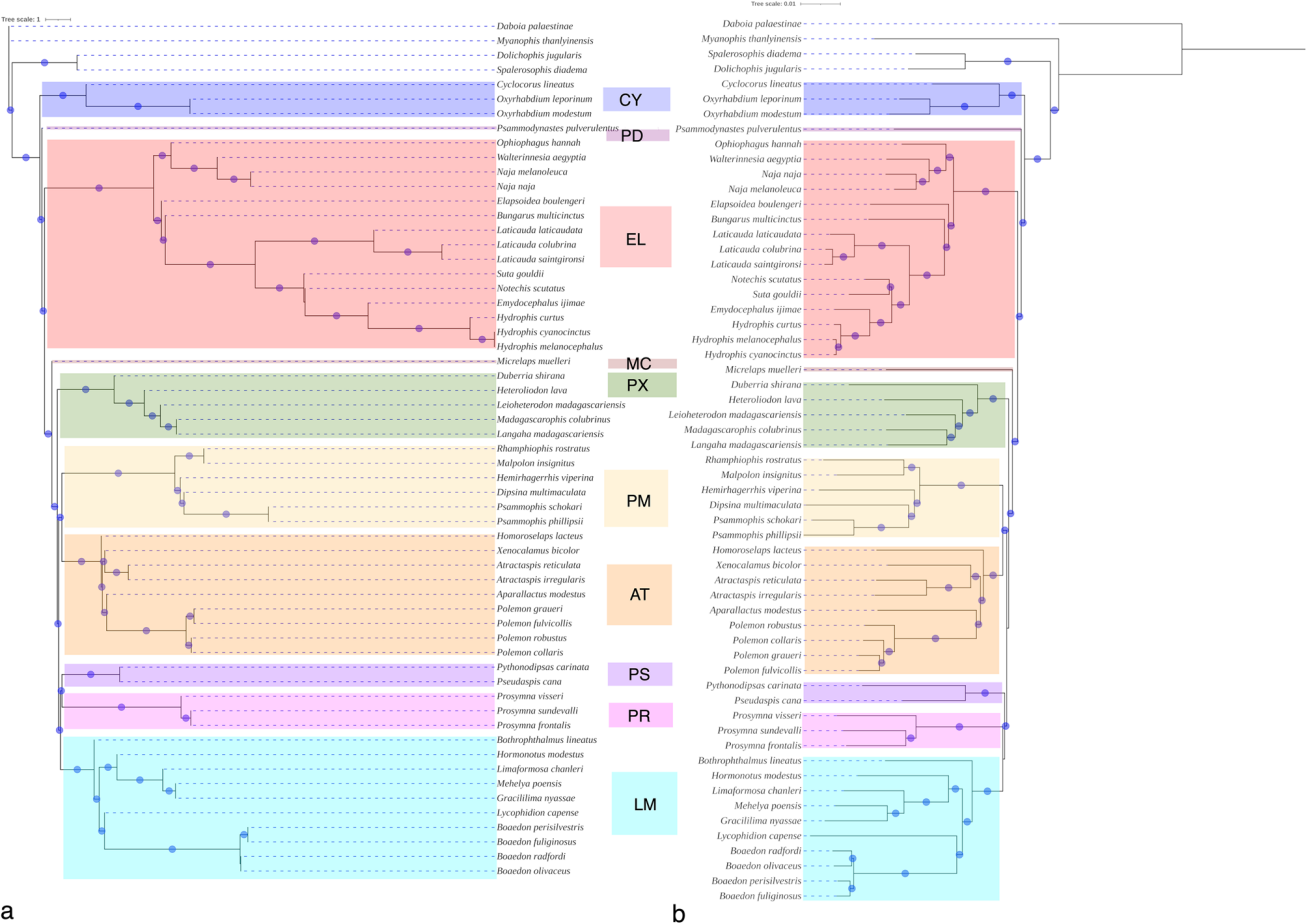
Weighted ASTRAL-hybrid multispecies coalescent phylogeny (**a**) and concatenation-based maximum likelihood phylogeny (**b**) from 50% complete ultraconserved elements dataset. Branches with a circle received > 0.95 local posterior probability or > 85% ultrafast bootstrap support. *AT* Atractaspidinae, *CY* Cyclocoridae, *EL* Elapidae, *LM* Lamprophiinae, *MC* Micrelapidae, *PD* Psammodynastidae new family, *PM* Psammophiinae, *PR* Prosymninae, *PX* Pseudoxyrhophiinae.

In all the UCE-only wASTRAL-h MSC phylogenetic trees, *Psammodynastes pulverulentus* represented a unique branch that is neither nested within nor sharing the most recent common ancestor with any of the recognised family group level taxa (Fig. 1a, Figs. S1–S3). This branch was the sister clade consisting of Elapidae, Lamprophiidae and Micrelapidae and this bipartition received a local posterior probability (localPP) of 1, the highest possible value.

**Figure 2:**
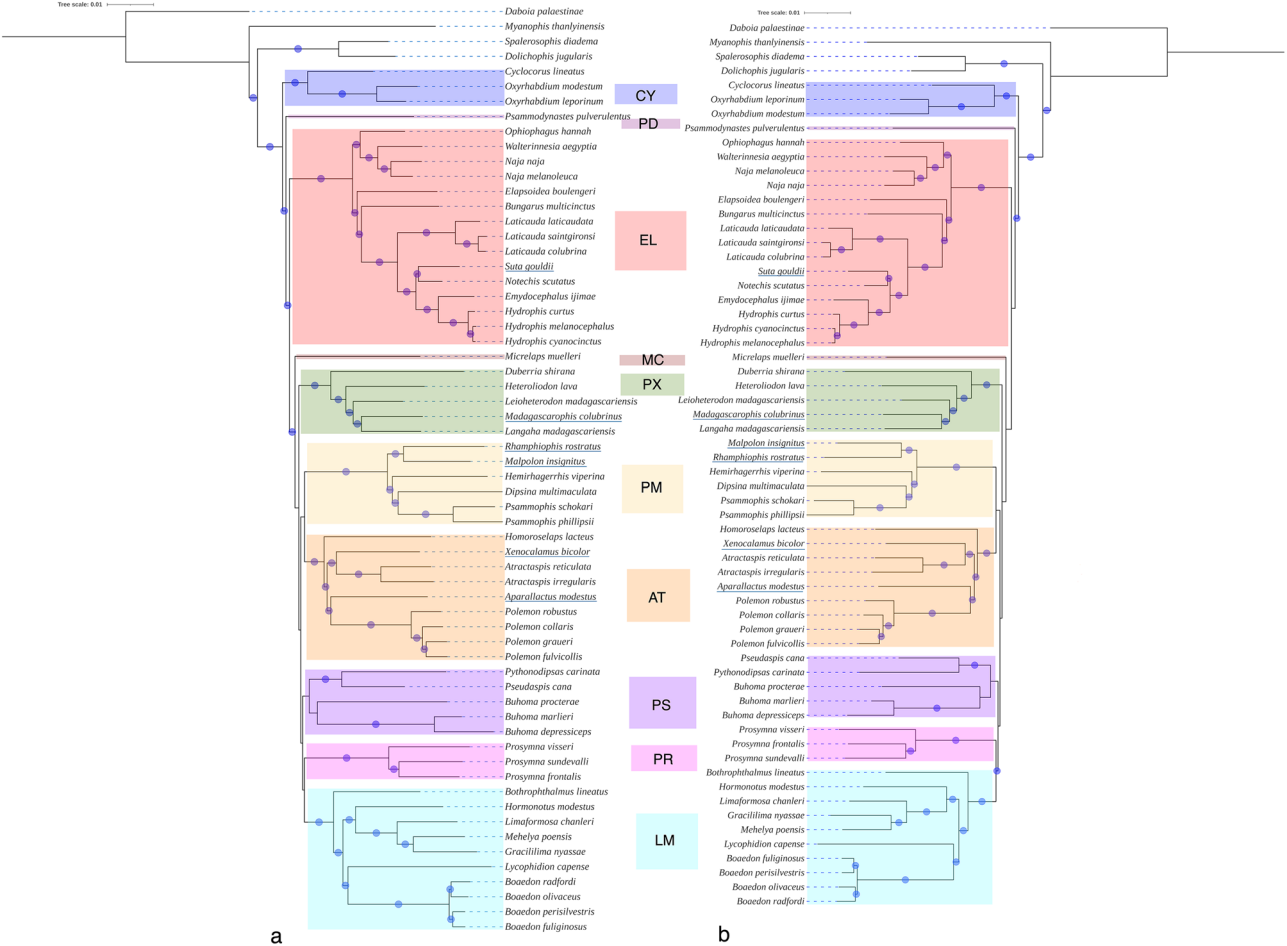
Concatenation-based maximum likelihood (**a**) and Bayesian inference (**b**) phylogeny from 95% complete ultraconserved elements dataset and traditional nuclear markers. Taxa with an underline are composites (in the traditional markers), though they are labelled with species contributing the largest data partition, i.e., UCEs, see supplementary table 1. Branches with a circle received > 0.95 posterior probability or > 85% ultrafast bootstrap support. Abbreviations as in Fig. 1.

In all the UCE-only and UCE + traditional nuclear marker concatenated data-based ML phylogenies (Fig. 1b, 2a, Figs. S4–S9), *P. pulverulentus* was recovered as sister to a clade comprising Elapidae, Lamprophiidae and Micrelapidae. UFBoot branch support values, computed by resampling from within partitions and sites within each partition to lessen the chances of getting artifactually inflated values, were 100 in all the UCE-only phylogenies. UFBoot values ranged from 98 to 93 in the UCE + traditional nuclear loci trees. The ExaBayes consensus topology recovered *P. pulverulentus* in the same position in the tree as in the ML topologies, with moderate posterior probability (PP) support of 0.86 (Fig. 2b, Fig. S13).

The position of the *Buhoma* spp., represented in our dataset only by Sanger-sequenced loci, was not resolved with significant branch support. In all the UCE + traditional nuclear marker ML and BI phylogenies, *Buhoma* genus was monophyletic (Fig. 2a, b, Figs. S7–S9). In the 50% complete UCE + traditional nuclear ML phylogeny, the common ancestral branch of *Buhoma* spp. formed a polytomy with two other clades, namely (Pseudaspidinae (Prosymninae, Lamprophiinae)) and (Atractaspidinae, Psammophiinae) (Fig. S7). In the ML and BI phylogenetic trees estimated from respectively 75% and 95% complete UCE plus traditional nuclear markers, *Buhoma* genus was sister to the Pseudaspidinae (Fig. 2a, b, Figs. S8, S9). The monophyly of *Buhoma* and the phylogenetic placements of this genus did not receive high UFBoot or PP support in any of the phylogenies. However, the sister taxon relationship between *B. depressiceps* and *B. marlieri* always received high UFBoot and PP branch support.

In the UCE + traditional mitochondrial and nuclear (mito-nuclear hereafter) loci ML phylogenetic trees, *B. depressiceps* + *B*. *marlieri* was sister to *P*. *pulverulentus*. The *P. pulverulentus* plus *B. depressiceps*-*marlieri* clade was sister to (Elapidae (Micrelapidae, Lamprophiidae)) (Figs. S10–S12). *Buhoma procterae* was sister to Prosymninae in the 95% complete UCE + traditional mito-nuclear loci phylogeny. In the 50 and 75% complete UCE + traditional mito-nuclear phylogenies *Buhoma* was sister to Pseudaspidinae. Barring the sister taxon relationship between *B. depressiceps* and *B. marlieri*, all the phylogenetic positions of *Buhoma* and *Psammodynastes* in the UCE + traditional mito-nuclear phylogenies were poorly supported.

We did not recover a monophyletic *Buhoma* in any of the traditional loci-only phylogenies (Figs. S14–S16). In the ML tree inferred from traditional nuclear markers, *B*. *procterae* was sister to *Psammodynastes* and these two were clustered with *depressiceps-marlieri*. These branches received UFBoot supports of ~ 85. This subclade itself was sister to Pseudaspidinae but with low support. In the traditional mito-nuclear phylogeny, a clade consisting of *P*. *pulverulentus* and *B depressiceps* + *B. marlieri* was the basalmost split within Elapoidea but *B. procterae* was clustered with Prosymninae in a more nested position in the tree (Fig. S16). In the mitochondrial phylogeny, neither *Psammodynastes* nor *B. depressiceps* + *B. marlieri* were recovered as members of Elapoidea but *B*. *procterae* is sister to Prosymninae within Elapoidea (Fig. S15).

Overall, the UCE-only topologies showed the same relationships between the major elapoid subclades as in^7^. The same held true for UCE + traditional nuclear and mito-nuclear loci topologies. The inferred generic interrelationships within Elapidae, whose taxon sampling increased from^7^, is well corroborated by other studies (e.g.,^3,5^). The traditional nuclear gene phylogeny inferred a monophyletic Lamprophiidae (with *Psammodynastes* in it) and at the deeper level a polytomy between Micrelapidae, Cyclocoridae and Elapidae + Lamprophiidae. Subfamilial interrelationships within Lamprophiidae in the traditional nuclear gene phylogeny differed from UCE trees. However, the mitochondrial and mito-nuclear topologies showed marked incongruence—this includes the placement of basal splits (assuming UCE tree to be ‘correct’), namely Cyclocoridae and Elapidae, as highly nested within Elapoidea and a failure to recover a monophyletic Lamprophiidae.

### Concordance factor, quartet support and reticulations

Site concordance factor (sCF) of the split between *Psammodynastes* and (Elapidae (Micrelapidae, Lamprophiidae)) clade was 33.95% in the UCE ML phylogeny (Fig. S17). The sCF value for other deep splits (i.e., between clades recognised at family/subfamily rank) in this phylogeny ranged from 33.82 to 54.99%. The quartet support for the phylogenetic position of *Psammodynastes* in the wASTRAL-h phylogeny was 38% (Fig. S20). The percentage falls within the range of the quartet support for other deep splits in the elapoid phylogeny.

In the legacy nuclear marker tree, the branch leading to *Buhoma procterae* and *P. pulverulentus* had an sCF of 45.72% (Fig. S18). The branch ancestral to *B. procterae* + *P. pulverulentus* and *B. depressiceps* + *B. marlieri* received an sCF of 55.08% whereas the clustering of this clade to Pseudaspidinae had a 26.58% sCF. In the mitochondrial marker phylogeny, the clustering of *B. procterae* with Prosymninae had 33.33% sCF whereas the clustering of *B. depressiceps* and *B. marlieri* with the colubrid outgroup received an sCF of 31.36% (Fig. S19).

**Figure 3:**
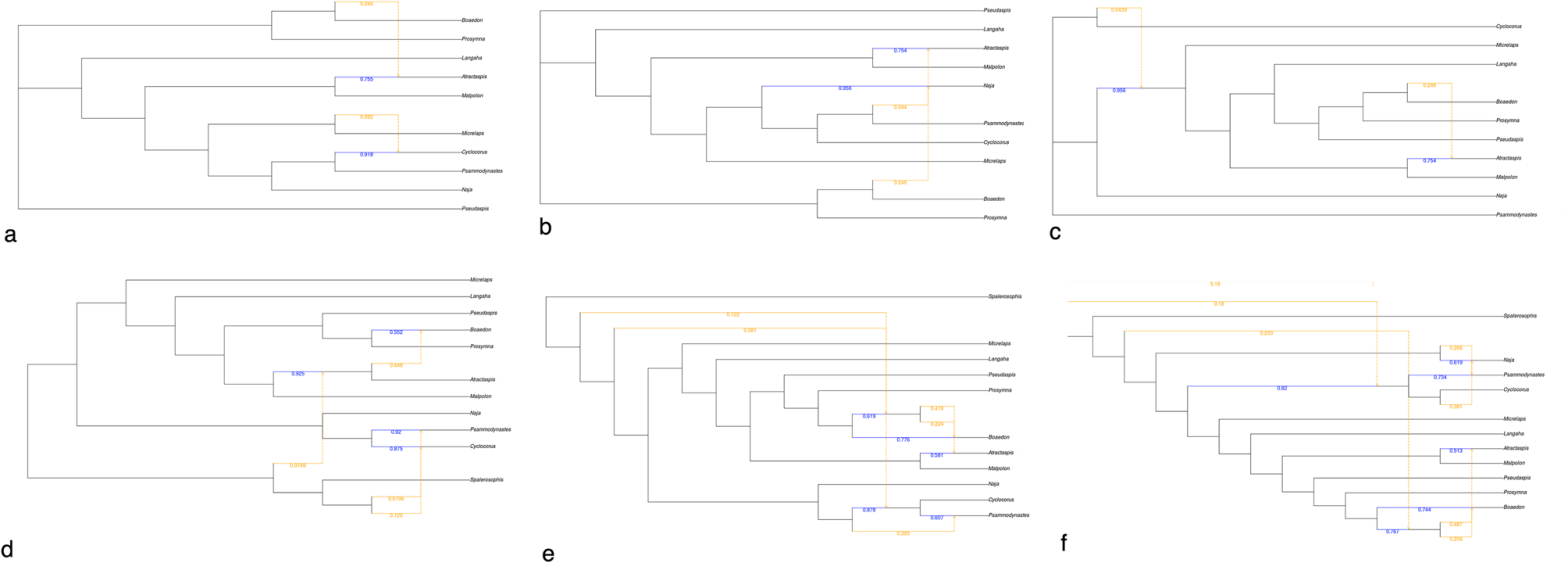
Multispecies coalescent phylogenetic networks with the best log-likelihoods, PhyloNetworks network with 2 (**a**), 5 (**b**) and 6 (**c**) permissible reticulations and PhyloNet network with 4 (**d**), 5 (**e**) and 6 (**f**) permissible reticulations. Major reticulation edges are blue and minor reticulation edges are orange. Values on the edges are the inheritance probabilities. Networks estimated with PhyloNetworks are unrooted.

Phylogenetic networks estimated with a maximum of 2, 5 and 6 permissible reticulations were the top three highest log-likelihood estimates in PhyloNetworks analyses, with 2 being marginally better than 5 and 6 (Fig. 3a–c, Fig. S22). None of these MSCNs had more than two inferred reticulations. A reticulation was between Lamprophiinae (represented by *Boaedon olivaceus*) and Atractaspidinae (represented by *Atractaspis reticulata*), with inheritance probability of the minor reticulation edge being ~ 0.25. Another reticulation event involving either Micrelapidae or Cyclocoridae (represented by *Cyclocorus*), Elapidae (represented by *Naja melanoleuca*) and *Psammodynastes* or a ghost introgression (i.e., an introgression from an extinct/unsampled taxon^12^) was estimated but this had a minor edge inheritance probability > 0.1.

PhyloNet MSCNs, on the contrary, inferred more horizontal edges with more permissible reticulations. MSCNs with 4, 5 and 6 permitted reticulations achieved the three best log-likelihoods, the one with 6 being the best by a narrow margin (Fig. 3d–f, Fig. S23). These networks too inferred a reticulation event involving Lamprophiinae or an extinct/unsampled sister and Atractaspidinae. The inheritance probability of this reticulation approached 0.5. The six reticulations MSCN had one 0.27 minor edge inheritance probability reticulation involving Elapidae and *Psammodynastes*. PhyloNet also inferred reticulation edges that did not originate from either the sampled extant lineages or their common ancestral branches. Such reticulations have been interpreted as ghost introgression, i.e., gene flow from an extinct lineage, in literature (e.g.,^25^). However, we refrain from interpreting these particular horizontal edges as ghost introgression for reasons provided in the Discussion.

### Timetree

**Figure 4:**
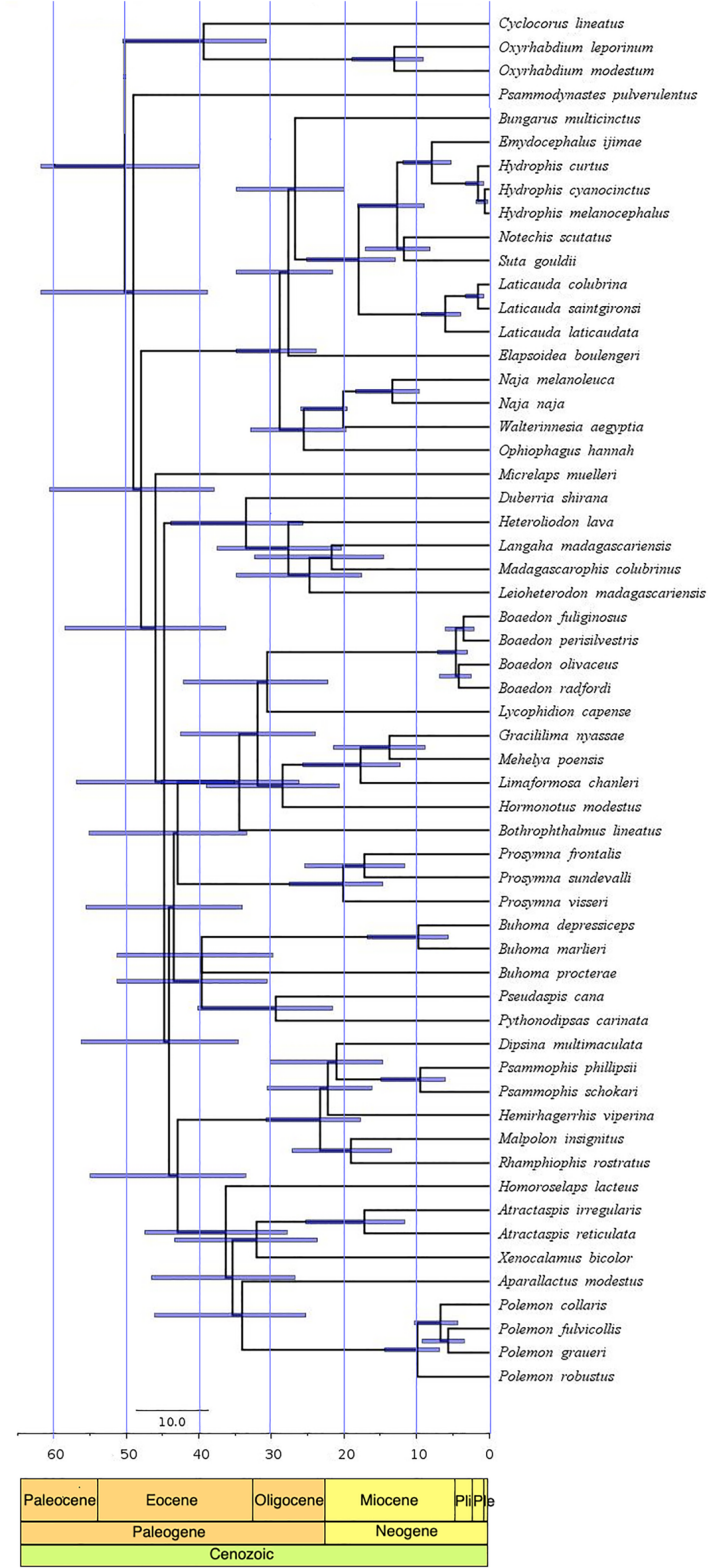
RelTime time-calibrated phylogeny of Elapoidea.

The divergence of *Psammodynastes* from a clade consisting of Elapidae, Lamprophiidae and Micrelapidae was dated at ~ 49 MYA (confidence interval 38.8–61.8 MYA) in the early Eocene (Fig. 4, Figs. S24, S25). The clade comprised of *Buhoma procterae*, *B. depressiceps* + *B. marlieri* and *Pseudaspis cana* + *Pythonodipsas carinatus* was dated as a polytomy at 39.6 MYA (confidence interval ~ 30 to 51.2 MYA), in the late Eocene whereas the divergence of *B. depressiceps* and *marlieri* was dated to be as recent as 9.7 MYA, the late Miocene. All the other divergence dates were close to those estimated by^7^ and confidence intervals also broadly overlapped.

### Cranial osteology

#### Psammodynastes

**Figure 5:**
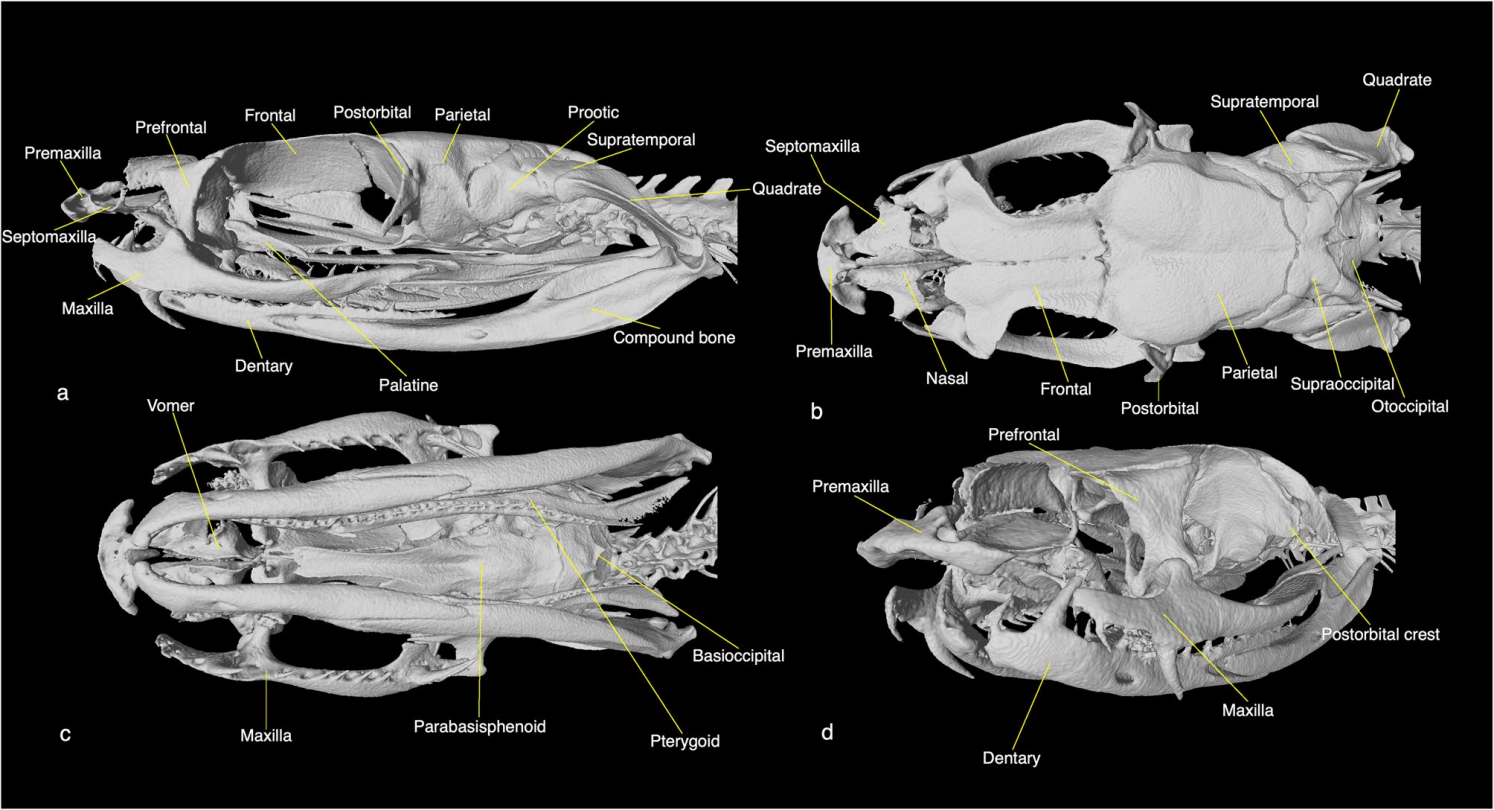
Lateral (**a**), dorsal (**b**), ventral (**c**) and anterolateral (**d**) views of the cranium of *Psammodynastes pulverulentus* (UMMZ:Herps 175728). Museum acronym: *UMMZ* University of Michigan Museum of Zoology.

The maxilla of *Psammodynastes pulverulentus* (n = 1; Table S6) is elongated, with distinct ascending, palatine and ectopterygoid processes (Fig. 5a, c). The maxilla bears small, ungrooved teeth in the beginning, then an ungrooved, elongated ‘fang’, then a series of small, ungrooved teeth and finally, enlarged, grooved rear-fangs. This maxillary dentition serves to distinguish this genus from all the elapoids examined by us except *Psammophis* spp.

Both the maxillary and the mandibular dentition of *Psammophis* are almost identical to those in *Psammodynastes*. *Psammodynastes* can be distinguished from *Psammophis* from the lack of a canthal ridge on the prefrontal lateral lamina and a moderate-sized optic foramen (Fig. 4b) (versus very large, lacertiform optic foramen of *Psammophis*).

Additionally, *Psammodynastes pulverulentus* possesses a very pronounced (nearly as wide as the orbital lamina of the parietal and as high as wide), squarish postorbital crest on the anterolateral aspect of the parietal bone for articulating the postorbital (Fig. 5b, d). This is a usual site of origin of the *levator anguli oris* muscle in colubroid snakes and very likely this crest also serves that purpose. Among the examined genera, only *Ophiophagus hannah* and *Notechis scutatus*, both belonging to Elapidae, possess a very well-developed, rounded or squarish postorbital crest. Elapid genera *Naja*, *Cyclocorus* and *Levitonius* of Cyclocoridae family, *Alluaudina*, *Compsophis*, *Langaha*, *Leioheterodon*, *Lycodryas* and *Madagascarophis* belonging to Pseudoxyrhophiinae and *Buhoma* can develop a distinct, narrow or triangular, tapering process from the ventral end of the postorbital ridge; this process, however, is very different from the large, squarish crest in *Psammodynastes*.

#### Buhoma

**Figure 6:**
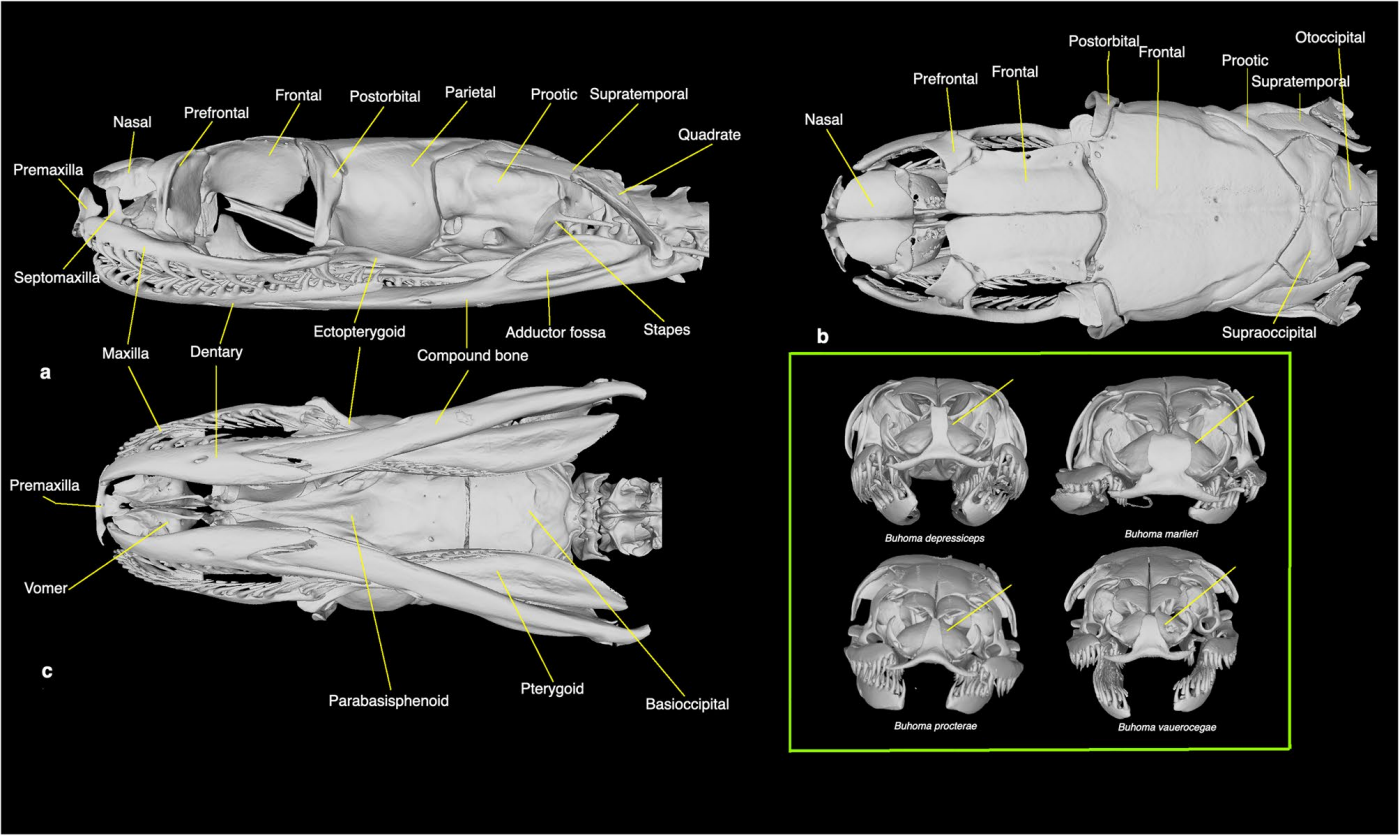
Lateral (**a**), dorsal (**b**) and ventral (**c**) views of the cranium of *Buhoma depressiceps* (BE-RBINS-VER-REP-16404). Inset—anterior view of *B. depressiceps* (BE-RBINS-VER-REP-16404), *B. marlieri* (BE-RBINs-VER-REP-8620), *B. procterae* (BE-RBINS-VER-REP-18541) and *B. vauerocegae* (BE-RBINS-VER-REP-18542), showing differences in the premaxillary ascending process (pointed at by the yellow line). Museum acronym: *RBINS* Royal Belgian Institute of Natural Sciences.

Unlike *Psammodynastes*, the *Buhoma* (n = 5; Table S6) cranium has few distinctive features that can serve to unequivocally distinguish this genus from other elapoids (Fig. 6a–c, Figs. S26–S29). All the examined *Buhoma* spp. have a large perforation, one-third to half the diameter of the lacrimal foramen, on their prefrontal lateral lamina. Studied elapoids, barring *Naja*, either lack such a foramen or possess only a very small one. However, this character seems somewhat variable within *Buhoma* itself, some specimens having two small foramina on one prefrontal lateral lamina and a large one on the other, and some *Naja* spp. possess a similar foramen. Apart from the elapoid subclades with unequivocal diagnostic features (viz., dentition in elapids, micrelapids, prosymnines, *Psammodynastes* and some psammophiine and atractaspidines or strong fossorial adaptation in the snout complex bones and braincase in prosymnines and some atractaspidines), *Buhoma* cannot be broadly diagnosed from any major elapoid subclade. There is no synapomorphy associating *Buhoma* with any recognized family or subfamily-level subclade.

*Buhoma marlieri* can be distinguished from all the other congenerics in having a wide, squarish, shield-like premaxillary ascending process, with a slightly constricted base and a slightly embayed or W-shaped upper margin (*versus* a narrow, rectangle-shaped ascending process in *B. depressiceps* and a triangular, dorsally tapering ascending process in *B. procterae* and *B. vauerocegae*). We did not find any cranial features clearly distinguishing *B. procterae* from *B. vauerocegae*.

## Discussion

Multispecies coalescent and concatenation-based approaches inferred highly congruent, well-supported phylogenies for Elapoidea and one of the focal genera, i.e., *Psammodynastes*, when the input dataset was UCE or UCE + legacy nuclear loci. However, there can be discordant signals in the underlying data (viz., incongruent gene trees) even with highly supported phylogenies. Such discordance may result from both biological and analytical factors^13^, such as incomplete lineage sorting (ILS), different types of data (viz., organellar versus nuclear genome), hybridisation and introgression, rogue taxa etc. Site concordance factor and quartet support for the best and the alternative topologies inferred with UCEs show the presence of a considerable amount of conflict in the data when it comes to the backbone of the phylogeny. The sCF value was close to 33% on several branches (Figs. S17, S20), including the split between *Psammodynastes* and the rest of the Elapoidea. Such sCF value is indicative of a paucity of decisive phylogenetic signal in favour of any particular resolution of quartet topologies in these parts of the tree and possibly also ILS^18^. These branches, including the common ancestor of *Psammodynastes* and the other elapoids, were also very short in terms of both the actual time and coalescent units. The problem of deep coalescence in rapid radiation, with a concentration of very short branches, is a well-known issue^7,14,15^. Therefore, both low phylogenetic signal and ILS might have affected these branches. Nevertheless, this was not a consistent pattern across the elapoid phylogeny as some very short branches (for instance, the earliest split of Cyclocoridae) had a high sCF value and quartet support.

Gene flow can give rise to conflicts similar to those caused by ILS^19^, necessitating a test of hybridisation and introgression prior to accepting the null hypothesis of ILS being the primary driver of discordance. Instances of hybridisation and introgression are often there in the history of rapid radiations (e.g.,^7,16^) and may even drive such explosive diversification^17^. With several reports of widespread reticulations in vertebrate phylogeny (e.g.,^20^), including even instances of actual hybrid speciation^21,22^, it is now evident that reticulation events have been widespread in the evolutionary history of vertebrates. The Maximum Pseudolikelihood networks estimated with PhyloNet and PhyloNetworks differ in the former preferring hypotheses with more reticulation events. The best networks of both methods inferred either a direct (both PhyloNet and PhyloNetworks) or ghost reticulation (only PhyloNet) event involving Lamprophiinae and Atractaspidinae. Lamprophiinae or an extinct or unsampled lineage sister to lamprophiines has been identified as the donor and Atractaspidinae as the recipient in most of the higher log-likelihood networks. Such a reticulation event is not biogeographically implausible considering both lamprophiines and atractaspidines are mostly distributed in sub-Saharan Africa. *Psammodynastes* was not implicated in any genetic inflow/outflow events with an inheritance probability equalling or exceeding 0.1 in PhyloNetworks analyses and PhyloNet reported only ghost introgression events or, with six permissible reticulations, an introgression event from Elapidae (Fig. 3e–f). Phylogenetic studies usually recover *Calliophis* as the earliest split within the Elapidae (e.g.,^3,6^), a genus with an Asian distribution like *Psammodynastes*. Therefore, it is possible that the stem lineages of both elapids and *Psammodynastes* coexisted in the same region and experienced some gene flow. However, there are serious caveats in these interpretations of the network analyses. Elapoidea is a radiation dating back to the Eocene and hence, it is very likely that several early major lineages are now extinct. Ghost introgression in the past from extinct lineages is particularly probable in such cases^12^. The presence of such unaccounted-for ghost introgression can significantly distort the result of many common tests of gene flow^23,24^. MPL network methods have gained popularity owing to being computationally efficient, unlike full likelihood or Bayesian approaches and minor reticulation edges from an interbranch to a terminal branch have routinely been interpreted as indicative of ghost introgression (e.g.,^23,25^). However, recent investigations with simulation data and reanalysis of published empirical data demonstrated that pseudolikelihood approaches are not capable of discerning ghost introgression from other plausible scenarios and the direction of gene flow estimated with such methods may not be reliable either^24^. Therefore, we caution against regarding the reticulation edges from ancestor to terminal branches in our networks as evidence of gene flow from unsampled, or more likely, extinct lineages. We further stress that care should be exercised with the donor-recipient directions in estimated reticulation events in the present study. We also refrain from interpreting some inheritance probabilities approaching 0.5 as suggestive of hybrid origin of a lineage because continuous gene flow can also result in such a scenario^26^. PhyloNetworks and PhyloNet disagree over these scenarios and phylogenetic networks in general are not designed to discern true hybridisation from other types of reticulation events^27^. A set of conservative conclusions would be that – 1. reticulation events probably occurred in the Afro-Malagasy radiation (Lamprophiidae), the stem lineages (or their extinct sister taxa) of Lamprophiinae and Atractaspidinae being the more likely contenders, 2. some relatively less probable reticulation events may involve early stem elapids, cyclocorids, *Psammodynastes* and possibly lineages that are now extinct.

Given that reticulation edges were present in phylogenetic networks, including the ones having the best log-likelihood, the null hypothesis that ILS solely explains all the conflicts in the backbone of the elapoid Tree of Life cannot be accepted. Also, not all the deep branches have been consistently implicated in reticulation events. Thus, ILS cannot be rejected as the principal factor (probably in conjunction with low phylogenetic signal) in producing discordant signal on those bipartitions.

Site concordance factors frequently went down below 33% for deep splits in traditional mito-nuclear loci phylogenies. Branch supports were also low in these phylogenies. The mitochondrial topology was especially dissimilar from those estimated with UCEs. Concatenation of mitochondrial loci with traditional nuclear markers and UCEs lowered the ultrafast bootstrap support throughout the backbone of the phylogeny and in the case of Sanger nuclear markers alone, changed the topology as well. Evolutionary geneticists have long treated mitochondrial genomic sequences separately from nuclear data, and this has resulted in identification of interesting evolutionary phenomena such as directional gene flow mediated by one sex (e.g.,^28^), possible despeciation/reverse speciation (e.g.,^29^) etc. In contrast, it has been the usual practice in phylogenetics to concatenate mitochondrial and nuclear sequences, even when the taxonomic group under investigation is an old radiation (e.g.,^1,4,6^) for which mitochondrial genes with fast substitution rates may not be a suitable phylogenetic marker. The obscuring of the already low phylogenetic signal on the short, deep edges by the mutational saturation on the long descendant edges is indeed an issue with resolving ancient, rapid radiations^14^. Mitochondrial genes can and probably do exacerbate this problem. Therefore, it seems advisable to treat mitochondrial and nuclear markers separately when studying old, rapid radiations.

Genus *Buhoma* is an interesting case; this genus was not monophyletic in the phylogeny of^4^ and in our UCE + traditional mito-nuclear marker phylogenies and the phylogenies computed only with traditional markers. These phylogenies also recovered some of the controversial placements of *Buhoma* spp. inferred in past studies, such as *Buhoma* as sister to *Psammodynastes* (as in^3^) or *Prosymna* (as in^4^), even though our taxon and gene sampling both were higher for this genus than those in previous studies. What can possibly explain these conflicts? The common ancestral branch of the three *Buhoma* spp. is short in the combined UCE-Sanger nuclear loci phylogenies. The timetree has a polytomy of *B. procterae*, *B. depressiceps* + *B. marlieri* and *Pseudaspis* + *Pythonodipsas* but the branches leading to *B. procterae* and *B. depressiceps* + *B. marlieri* are long. It therefore appears that *Buhoma* itself could be a case of ‘ancient, rapid radiation’. Rapid diversification soon after the origin of the stem *Buhoma* might have resulted in the accumulation of little molecular and morphological synapomorphies that could help cluster them in a cladistic analysis. Millions of years of evolution on the long descent lineages then possibly led to mutational saturation which caused further obfuscation of any meaningful phylogenetic signal. Lack of decisive support for monophyly in small scale molecular data, combined with a potentially misleading ‘signal’ (phylogenetic noise) causes some or all the *Buhoma* spp. to behave as rogue taxa. The association of *Buhoma* with *Psammodynastes* is also possibly caused by the same factor. Homoplasy most probably resulted in clustering of one or two *Buhoma* spp. with *Psammodynastes*. In the absence of genome-wide data, this resulted in a more nested position for *Psammodynastes* in the phylogeny, i.e., within Pseudaspidinae (as in^3^ and our traditional marker trees). When combined with UCE data, the position of *Psammodynastes* was correctly inferred but this had the effect of pulling some *Buhoma* spp. down the tree when the mitochondrial partition was added. Based on these considerations and biogeography, we regard the association of *Buhoma* with *Psammodynastes* as an artefact of inadequate data and homoplasy. One theoretical expectation of rogue taxa is that by being able to get placed almost anywhere in the phylogeny, such taxa lower the overall branch support throughout the tree^5^. The marked lowering of branch support in the UCE + Sanger sequenced mito-nuclear loci tree may be a combined effect of both highly conflicting signals from the mitochondrial genes and the rogue behaviour of *Buhoma*. Some phylogenetic problems are resolvable only with either whole or reduced representation genome-scale data^30^ and *Buhoma* seems to be one such problem. However, there is support, albeit weak, in the nuclear genes to unite *Buhoma* with Pseudaspidinae and therefore, we provisionally regard *Buhoma* as a member of Pseudaspidinae, as was done by Pyron et al.^3^. It is noteworthy that the divergence between *B. depressiceps* and *B. marlieri* is older than that between several other species pairs in our timetree (Fig. S24). This and the osteological difference in the premaxilla support the decision of Chippaux and Jackson^31^ to treat *B. marlieri* as a separate species.

The phylogenetic position of the mock vipers, *Psammodynastes*, has been well-resolved and consistent across methods when using target capture data in the present study. There are also distinct apomorphic character states diagnosing this lineage from other elapoid subclades. *Psammodynastes* is the sister taxon to the common ancestral branch of Elapidae, Micrelapidae and Lamprophiidae and is not nested within any family group taxon. Therefore, the only Linnean rank appropriate for it would be a family. There are no available suprageneric nomina to which this genus was exclusively assigned in the past. Hence, we describe Psammodynastidae *new family*, belonging to the Elapoidea superfamily, to accommodate *Psammodynastes*:

Psammodynastidae *new family*

ZooBank LSID: urn:lsid:zoobank.org:pub:1F332821-51B7-4E47-9FBD-84E79E3269EB

Type genus: *Psammodynastes* Günther, 1858

Type species: *Psammodynastes pulverulentus* (Boie, 1827)

Etymology: The generic name is a combination of ancient Greek word *psammos* (ψάμμος, meaning sand) and *dynastes* (δυνάστης, meaning ruler). We derive the family name by adding -idae to the stem of the generic suffix.

Contents: *Psammodynastes pulverulentus* (Boie, 1827), *P. pictus* Günther, 1858

Diagnosis and definition: Maxilla of *Psammodynastes* bears 2 ungrooved, small teeth at the anterior end, followed by 1–2 enlarged, ungrooved teeth (occasionally with several shallow, non-venom carrying grooves^32^), then 5–9 small, ungrooved teeth and finally, 2 enlarged, grooved rear fangs (present study^10,33^). The mandible bears enlarged teeth at the anterior end (present study^33^). The *Psammodynastes* cranium has a very well-developed postorbital crest from the parietal. Vertebrae of *Psammodynastes* bear hypapophyses throughout^10^. The combination of—I. maxillary dentition, II. pronounced postorbital crest, III. prefrontal bone devoid of a canthal ridge and IV. moderate-sized optic foramen (i.e., frontal orbital laminae contact the crest on the parasphenoid cultriform process for at least two-thirds of the length of the latter) distinguishes Psammodynastidae *new family* from all other elapoid families and subfamilies.

In external appearance, the head is distinct from the neck and has a canthal ridge (not to be confused with the canthal ridge on the prefrontal bone in Psammophiinae) anterodorsal to a large eye with a vertical pupil. There are 8–9 supralabials, of which the 3rd to 5th enter the orbit, 8–9 infralabials, and the temporal formula is 2 + 2 or 2 + 3^8,33^. Dorsal scales are smooth, devoid of apical pits and there are usually 17 (rarely 19) dorsal scale rows at midbody^8,10,33^. There are 146–175 ventrals, a single anal plate and 44–88 paired subcaudal scales^8^.

Distribution: South Asia (North-eastern India, Nepal), southern China, Southeast Asia (Myanmar, Thailand, Malaysia, Indonesian archipelago, Cambodia, Laos, Vietnam, Philippines) and Taiwan.

## Materials and methods

### Genome sequencing, processing of target capture and whole genome sequence data

The ultraconserved elements (UCE) of 47 taxa, including 44 elapoids and three of the four outgroups, are from Das et al.^7^. These samples were specifically enriched and sequenced for UCEs^7^.

UCEs of 11 more elapoids, including one of the focal taxa of this paper—*Psammodynastes pulverulentus*, and ten elapids, and one additional outgroup taxon belonging to Homalopsidae, *Myanophis thanlyinensis*, were harvested in silico from whole genome sequences. The reference genome sequencing of *P. pulverulentus* and assembly were performed at Iridian Genomes (Bethesda, USA). Sequencing of reference genome, described in this section, and Sanger sequencing and CT scans, reported under other sections, utilised only preserved museum specimens and no live animals were collected/experimented upon/euthanised for our study. Genomic DNA from a *P*. *pulverulentus* (FMNH 273629) from the collection at the Field Museum of Natural History (Chicago, USA) was extracted with the Qiagen DNAEasy kit following the standard instructions. Library preparation was done using Illumina TruSeq kit with standard adapters and following the standard protocol. Sequencing at 105X coverage was carried out on llumina X-Ten platform. Raw reads were preassembled to scaffold (103,289 scaffolds) level with SPAdes version 3.15.4^34^ and Zanfona (https://github.com/zanfona734/zanfona) was used for genome finishing. The assembly has been made available on the NCBI Genome database (https://www.ncbi.nlm.nih.gov/datasets/genome/GCA_025802295.1/). Rest of the genome assemblies were obtained from the NCBI Genome database (NCBI Taxonomy since July, 2023). Reference assembly name, link and associated publications, if any, for all the reference genomes, including that of *P. pulverulentus*, are listed in the Supplementary Information.

Reference genome sequences for all the elapoids and a homalopsid available in the NCBI Genome (NCBI Taxonomy) database were downloaded. The assembly of *Hydrophis hardwickii* has assembly contamination and hence, was not incorporated in the downstream analyses. The phyluce pipeline, version 1.7.2,^35^ was used to match the Tetrapods-UCE-5Kv1 bait set (https://www.ultraconserved.org/) to the genome sequences and create a database using the phyluce_probe_run_multiple_lastzs_sqlite function. Using the lastz database generated in that step, loci matching the UCE probe were extracted with the phyluce_probe_slice_sequence_from_genomes function, retaining 400 bp of downstream and upstream flanks. The outputs were symlinked to the contigs generated from the dataset of Das et al.^7^. To generate the necessary probe match database and configuration files for the downstream processing, the probe matching step was repeated with phyluce_assembly_match_contigs_to_probes command with the same Tetrapods-UCE-5Kv1 probe set. Finally, the phyluce_assembly_get_match_counts and the phyluce_assembly_get_fastas_from_match_counts functions were used to extract the UCEs for all the 59 taxa. MAFFT^36^ was used, in conjunction with edge trimming, to align each individual UCEs locus dataset on phyluce. Preparation of the final UCE multiple sequence alignments for phylogenomic analyses was carried out in the phyluce pipeline and includes three steps, namely, removal of UCE locus name from the taxon nomen, preparing datasets of three different levels of completion (viz., 50, 75 and 95% complete datasets) and generating concatenated datasets for combined data analyses.

### Sanger sequencing and preparation of Sanger sequence data

For this study, we have sequenced mitochondrial cytochrome b (*CYTB*), NADH dehydrogenase subunit 4 (*ND4*) and 16S ribosomal RNA gene (*16S*) and nuclear brain-derived neurotrophic factor (*BDNF*), oocyte maturation factor mos (*C-MOS*) and recombination activating gene 1 (*RAG1*) of *Buhoma depressiceps* (UTEP 22599) and *B. marlieri* (UTEP 22598) specimen in the collection of the University of Texas at El Paso (El Paso, USA). The PCR and sequencing primers for *BDNF* were as in^37^. The rest of the primers were from^38^. The laboratory protocols, including those for genomic DNA extraction, PCR amplification and Sanger sequencing follow^38^.

The raw Sanger sequencing reads were assembled using the Pearl programme of the Tracy toolkit^39^ hosted at GEAR-GENOMICS (https://www.gear-genomics.com/). The assembly was visually checked in Pearl and base calls were rectified, based on thorough inspection of the chromatogram, if needed. The *ND4* and *CYTB* sequences of *B. marlieri* were of low quality and hence, those were discarded from subsequent analyses. Open Reading Frames (ORF) of the protein-coding sequences were detected with NCBI ORF Finder (https://www.ncbi.nlm.nih.gov/orffinder/), with setting the ORF start codon as any sense codon. Afterwards, we performed a megablast on NCBI BLAST with default parameters to confirm the identity of the sequences we generated.

We searched for and obtained sequences of mitochondrial *CYTB*, *ND4*, *16S* and *12S* (12S ribosomal RNA gene) and nuclear *BDNF*, *C-MOS*, *RAG1* and *RAG2* (recombination activating gene 2) for elapoids and outgroups for which we have UCE data from NCBI GenBank. While most of the Sanger sequences in our dataset belong to the same species as in the UCE dataset, in a few instances we have included the sequence of a closely related congener if the sequence of a gene for a particular taxon is unavailable. This was done only if the taxon is the sole representative of its genus in our dataset and the congeneric status of the replacement is taxonomically uncontroversial. GenBank accession numbers of the sequences, including the ones sequenced by us for this study, and any other associated information are provided in the Supplementary Information (Table S1).

We do not have tissue sample of *Buhoma procterae* and therefore used *CYTB*, *ND4*, *12S*, *16S*, *C-MOS* and *RAG2* sequences from^11^. We also obtained *12S* and *RAG2* sequences of *B. depressiceps* generated in the same study. The *B. depressiceps* specimen used in that work originated from Gabon and hence, is unambiguously referable to *B. depressiceps* and not *B. marlieri*.

For taxa with a reference genome, we harvested nuclear genes from the genome assembly itself to alleviate the need for creating composites. To do this, we BLAST-ed conspecific/congeneric/confamilial sequences of the four nuclear loci separately against a genome with megablast and default parameters. The matched region was extracted and megablast-ed against the GenBank database, irrespective of whether the genome was annotated or not, to ensure that we have got the exact gene we were looking for. Nuclear loci sequences harvested in this way were added to our Sanger loci dataset. For the query sequence accession number, see Supplementary Information (Table S2–S5).

Sequences were aligned with MUSCLE^40^, implemented on MEGA 11^41^, with default parameters. Alignments were scrutinised visually. Overhangs of a few very long sequences at the ends that were missing in other sequences were trimmed. External gaps were coded as missing data with SequenceMatrix 1.9^42^. SequenceMatrix 1.9 was used to concatenate the Sanger loci. We have prepared the following concatenated datasets—(1) four nuclear loci, (2) four mitochondrial loci, (3) all eight Sanger loci, (4) four nuclear loci plus 50, 75 and 95% complete, concatenated UCE datasets, and (5) all eight Sanger loci plus 50, 75 and 95% complete, concatenated UCE datasets, using SequenceMatrix 1.9. Prior to combining Sanger nuclear and UCE loci, we ran local blastn (blast-2.10.0 +) of unaligned multifasta query of Sanger nuclear loci, with e-values 1e^−50^ and 0.05, against a database (prepared with makeblastdb [blast-2.10.0 +]) of unaligned multifasta of UCEs to ensure that UCEs flanks did not overlap any of the four nuclear genes. No match was detected in the local blastn searches.

### Phylogenomic analyses

We inferred quartet-based multispecies coalescent species trees from gene trees of individual UCE loci, maximum likelihood (ML) phylogenies from concatenated UCE only dataset, concatenated UCE + traditional marker dataset and combined traditional marker dataset and Bayesian Inference (BI) phylogeny from concatenated UCE + traditional marker dataset.

For the multispecies coalescent (MSC) phylogenetic tree estimation, we used weighted ASTRAL hybrid (wASTRAL-h) method^43^. Algorithms of weighted ASTRAL class weigh quartets based on branch support, branch length or both without requiring any arbitrarily specified threshold. This has been demonstrated to result in phylogenies more accurate than those produced by ASTRAL-III algorithm^43^. Among the three different weighting approaches, the one that weighs based on both the branch length and support (i.e., wASTRAL-h) performs the best. ML gene trees were estimated for 50, 75 and 95% complete UCE loci set with IQ-TREE 2.2.0.5^44^ with 1000 ultrafast bootstrap^45^ replicates per gene tree. Model selection for each locus was performed with the default ModelFinder^46^ option. MSC species tree was then inferred from the gene trees set with wASTRAL-h with default settings.

We used IQ-TREE 2.2.0.5 to infer ML species tree from the UCE-only concatenated matrices of 50, 75 and 95% completeness. The default model selection and optimum partitioning scheme finding algorithm of IQ-TREE, i.e., ModelFinder plus partition merging (MPF + MERGE option), proved to be computationally infeasible on our cluster. Hence, we chose the TESTMERGE option, which functions identically to PartitionFinder^47^ and is less computationally taxing. TESTMERGE differs from ModelFinder in that it does not consider the FreeRate heterogeneity model^46^ implemented in the latter. Additionally, we used the relaxed hierarchical clustering method^48^ to consider the top 10% most similar partition (individual UCE locus) pairs (by setting -rcluster to 10) which are then merged until no better partition merging schemes can be estimated for further computational efficiency. Branch support was assessed with 1000 ultrafast bootstrap (UFBoot) replicates. To reduce the possibility of wrong splits receiving high UFBoot support^49^, we resampled from within the partitions and sites within a partition by setting the –sampling to GENESITE.

We inferred ML phylogenies with UFBoot branch support from UCE + all eight Sanger loci and UCE + only the Sanger nuclear loci combined datasets with the same software and parameter settings as with the UCE-only ML analyses.

ML phylogenies were also inferred from concatenated Sanger-sequenced mito-nuclear, mitochondrial and nuclear loci datasets separately with IQ-TREE. Small sizes of these datasets allowed a full MFP + MERGE model selection and greedy search for the best partitioning scheme. For protein-coding genes, partitions were inputted as individual codon positions. UFBoot support, with within-partition and sites-within-partition resampling enabled, was computed for these phylogenies in the same manner as the UCE and UCE + traditional marker ML analyses.

Finally, we also computed a BI phylogeny from the 95% complete UCE and traditional nuclear loci dataset with ExaBayes 1.5.1^50^ to test if BI corroborates the result from the ML analyses of UCE and traditional marker combined data. We ran the MPI (message passing interface) version ExaBayes 1.5.1, i.e., exabayes, on unpartitioned UCE and nuclear loci matrix with 200 MPI processes. Two independent runs, each with four coupled chains (one cold, three heated), were run as parallel processes. Number of swap attempts per generation was set to 2. Sampling frequency was set to 500 generations. Diagnostics for convergence were checked every 5000 generations. Other parameters were left at default settings. Though the number of generations was set to 100 M prior to the commencement of the analysis, two consecutive runs (a second one from the checkpoint of the timed-out first run on the cluster) could reach only 2.7 M generations. However, the average standard deviation of the split frequencies (ASDSF), the default divergence metric of ExaBayes, fell to 3.88% which is lower than the default 5% convergence diagnostic value, thus indicating an acceptable level of convergence. A majority rule consensus topology was estimated from the posterior distribution of trees after discarding the first 25% as burn-in with the consense utility of ExaBayes.

All the phylogenomic analyses were run on the Puhti supercomputing cluster of the Finnish CSC-IT Centre for Science Ltd. Phylogeny visualisation was done with iTOL 6.7.6^51^.

### Analyses of conflict in data and topology

To better identify the potential causal factors that complicate the inference of the rapid radiation of elapoids, including the focal taxa, we investigated site concordance factor, quartet support and tested for the presence of any deep reticulation events.

We have computed the site concordance factor (sCF)^18^ for the 50% complete UCE wASTRAL-h and IQ-TREE species trees, traditional nuclear loci phylogeny and the mitochondrial marker-based phylogeny with IQ-TREE 2.2.0.5. ASTRAL 5.7.8^52^ was used to compute the quartet support for branches in 50% complete UCE wASTRAL-h phylogeny.

We prepared a 100% complete UCE dataset consisting of a colubrid outgroup (*Spalerosophis diadema*), one of the focal genera, namely *Psammodynastes*, and one representative taxon from every other elapoid families/subfamilies. This 11-taxon dataset was prepared with the phyluce pipeline. A set of unrooted ML gene trees was estimated with IQ-TREE 2.2.0.5 with the same parameters as with the gene trees estimated for wASTRAL-h analyses in the previous section. Another set of ML gene trees, rooted by specifying the outgroup with -o command, was also inferred in the same manner. The unrooted gene trees were used as input to PhyloNetworks^53^ in Julia 1.9.1 to estimate the quartet concordance factors (quartetCF). The quartetCFs were used to infer Maximum Pseudolikelihood (MPL) multispecies coalescent networks (MSCN) with the snaq! algorithm^54^ of PhyloNetworks. Firstly, one MSCN with zero reticulations (hence, essentially a tree) was inferred with the quartetCFs from the unrooted gene trees and a wASTRAL-h phylogeny computed from the same set of gene trees. For the subsequent MSCN estimation, with 1 to 6 reticulations (hmax), we used the gene trees set and the first MSCN (hmax = 0) as input. The best number of hybridisations was determined by plotting the log-likelihoods from the aforementioned runs against the hmax following the recommendation in the online manual. A second set of MPL-based MSCNs, with 0–6 reticulations, was estimated from the rooted gene trees with PhyloNet 3.8.2^55^. Unlike the snaq! of PhyloNetworks, the MPL algorithm of PhyloNet uses rooted triplets induced by the gene trees to compute the MSCN^56^. This method produced five MSCN per analysis. The best number of reticulations was determined by plotting the best log-likelihood from each analysis against the number of reticulations following^57^.

All the aforementioned analyses were conducted on a local machine. All the MSCNs were visualised with the PhyloPlots package on Julia 1.9.1.

### Timetree analysis

We estimated a time-calibrated phylogeny with the 95% UCE plus traditional nuclear loci dataset using the RelTime-ML^58,59^ as implemented on MEGA 11^41^. Log-normal calibration densities^60^ used were the same as in^7^. We set GTR + G + I as the substitution model for the analysis. The resulting Timetree was visualised with FigTree 1.4.4^61^.

### Micro-CT scan and comparison of anatomical data

We have μ -CT scanned the heads of *Buhoma depressiceps*, *B. marlieri*, *B. procterae* and *B. vauerocegae* in the holdings of the Royal Belgian Institute of Natural Sciences (Brussels, Belgium) and the Royal Museum for Central Africa (Tervuren, Belgium). These include the holotype of *B. marlieri* (RMCA-VER-REP 18091). The μ-CT scan of the cranium of *Psammodynastes pulverulentus* was obtained from MorphoSource. We also scanned four elapids, five lamprophiines, four psammophiines, one pseudaspidine and two prosymnines for comparison. We also examined additional μ-CT scans studied by^7^ to broaden the scope of the comparison. The μ-CT scans generated in the present study have been uploaded to MorphoSource and are listed in the Supplementary Information (Table S6).

Several specimens were scanned at the μCT facility of the Royal Belgian Institute of Natural Sciences. Depending on the size of the specimen, scans were performed using: a) an EasyTom 150 (RX Solutions, Chavanod, France) with an aluminum filter at 10–30 W, 110 kV, 5.5–12.5frames/s, 1440 projections per rotation and 6–26 μm isotropic voxelsize; b) an XRE UniTom (Tescan XRE, Ghent, Belgium) at 10–22 W, 75 kV, 150–400 ms frame rate, 1800 projections per rotation, and 6 to 11 μm isotropic voxelsize. Segmentation of the scans was done using Dragonfly software, Version 4.1 for Windows (Object Research Systems (ORS) Inc., Montreal, Canada, 2020, https://www.theobjects.com/dragonfly/index.html). Cleaning of the resulting 3D models was done using GOM Inspect (https://www.gom.com/). Terminology used for cranial bones and muscle attachment sites follows^62,63^.

### Supplementary Information

Supplementary Information.

## Data Availability

Ultraconserved elements, traditional nuclear and mitochondrial marker sequence datasets and the best partitioning schemes for the concatenation-based analysis of each dataset are available at https://figshare.com/account/home#/projects/173346. Micro-CT scans, Sanger sequences and the reference genome sequence generated for this study are uploaded to MorphoSource, NCBI Nucleotide and Genome databases respectively and the relevant ark, accession number and assembly names and links are listed and/or provided in the Supplementary Information.
