# Supplementary material for: Phylogenomics of Psammodynastes and Buhoma (Elapoidea: Serpentes), with the description of a new Asian snake family
Source: Sci Rep. 2024 Apr 25;14:9489. doi: 10.1038/s41598-024-60215-2 (PMC11045840; doi:10.1038/s41598-024-60215-2)
Supplement: Supplementary file 1 — Supplementary Information. [file 41598_2024_60215_MOESM1_ESM.pdf]

Supplementary Information

Das, S., Greenbaum, E., Brecko, J., Pauwels, O. S. G., Ruane, S., Pirro, S. & Merilä, J. Resolving the elapoid phylogeny: systematics of *Psammodynastes* and *Buhoma* (Elapoidea: Serpentes), with the description of a new Asian snake family

Reference genomes

The reference genomes listed here were used to harvest ultraconserved elements (UCE) loci and traditional nuclear markers *in silico*.

Bungarus multicinctus

Genome assembly: ASM2365372v1,

https://www.ncbi.nlm.nih.gov/datasets/genome/GCA_023653725.1/

Publication: Xu *et al*. Genomic, transcriptomic, and epigenomic analysis of a medicinal snake, *Bungarus multicinctus*, to provides insights into the origin of Elapidae neurotoxins. *Acta Pharm. Sin. B*, **13**, 2234–2249 (2023).

Daboia siamensis

Genome assembly: ASM2444931v1,

https://www.ncbi.nlm.nih.gov/datasets/genome/GCA_024449315.1/

Publication: Saethang *et al*. Identification of *Daboia siamensis* venome using integrated multi-omics data. *Sci. Rep*., **12**, 13140 (2022).

Remark: Only used for harvesting some traditional nuclear loci. UCEs for *D. palaestinae* were already available from [7].

Emydocephalus ijimae

Genome assembly: emylji_1.0,

https://www.ncbi.nlm.nih.gov/datasets/genome/GCA_004319985.1/

Publication: Kishida *et al*. Loss of olfaction in sea snakes provides new perspectives on the aquatic adaptation of amniotes. *Proc. R. Soc. B*, **286**, 20191828 (2019).

Hydrophis cyanocinctus

Genome assembly: HCya_v2,

https://www.ncbi.nlm.nih.gov/datasets/genome/GCA_019473425.1/

Publication: Li *et al*. Two Reference-Quality Sea Snake Genomes Reveal Their Divergent Evolution of Adaptive Traits and Venom Systems. *Mol. Biol. Evol.*, **38**, 4867–4883 (2021).

Hydrophis curtus

Genome assembly: HCur_v2,

https://www.ncbi.nlm.nih.gov/datasets/genome/GCA_019472885.1/

Publication: Li *et al*. Two Reference-Quality Sea Snake Genomes Reveal Their Divergent Evolution of Adaptive Traits and Venom Systems. *Mol. Biol. Evol.*, **38**, 4867–4883 (2021).

Hydrophis melanocephalus

Genome assembly: hydMel_1.0,

https://www.ncbi.nlm.nih.gov/datasets/genome/GCA_004320005.1/

Publication: Kishida *et al*. Loss of olfaction in sea snakes provides new perspectives on the aquatic adaptation of amniotes. *Proc. R. Soc. B*, **286**, 20191828 (2019).

Laticauda colubrina

Genome assembly: latCor_2.0,

https://www.ncbi.nlm.nih.gov/datasets/genome/GCA_015471245.1/

Publication: Kishida *et al*. Loss of olfaction in sea snakes provides new perspectives on the aquatic adaptation of amniotes. *Proc. R. Soc. B*, **286**, 20191828 (2019).

Laticauda laticaudata

Genome assembly: latLat_1.0,

https://www.ncbi.nlm.nih.gov/datasets/genome/GCA_004320025.1/

Publication: Kishida *et al*. Loss of olfaction in sea snakes provides new perspectives on the aquatic adaptation of amniotes. *Proc. R. Soc. B*, **286**, 20191828 (2019).

Myanophis thanlyinensis

Genome assembly: ASM1765603v1,

https://www.ncbi.nlm.nih.gov/datasets/genome/GCA_017656035.1/

Publication: Köhler *et al*. A new genus and species of mud snake from Myanmar (Reptilia, Squamata, Homalopsidae). *Zootaxa*, **4915**, 301–325(2021).

Naja naja

Genome assembly: Nana_v5,

https://www.ncbi.nlm.nih.gov/datasets/genome/GCA_009733165.1/

Publication: Suryamohan *et al*. The Indian cobra reference genome and transcriptome enables comprehensive identification of venom toxins. *Nat. Genetics*, **52**, pages106–117 (2020).

Notechis scutatus

Genome assembly: TS10Xv2-PRI,

https://www.ncbi.nlm.nih.gov/datasets/genome/GCF_900518725.1/

Publication: No specific publication (but the genome assembly has been used and cited by subsequent researches).

Ophiophagus hannah

Genome assembly: OphHan1.0,

https://www.ncbi.nlm.nih.gov/datasets/genome/GCA_000516915.1/

Publication: Vonk *et al*. The king cobra genome reveals dynamic gene evolution and adaptation in the snake venom system. *Proc. Natl. Acad. Sci*., **10**, 20651–20656 (2013).

Psammodynastes pulverulentus

Genome assembly: ASM2580229v1,

https://www.ncbi.nlm.nih.gov/datasets/genome/GCA_025802295.1/

Publication: This paper.

Supplementary figures

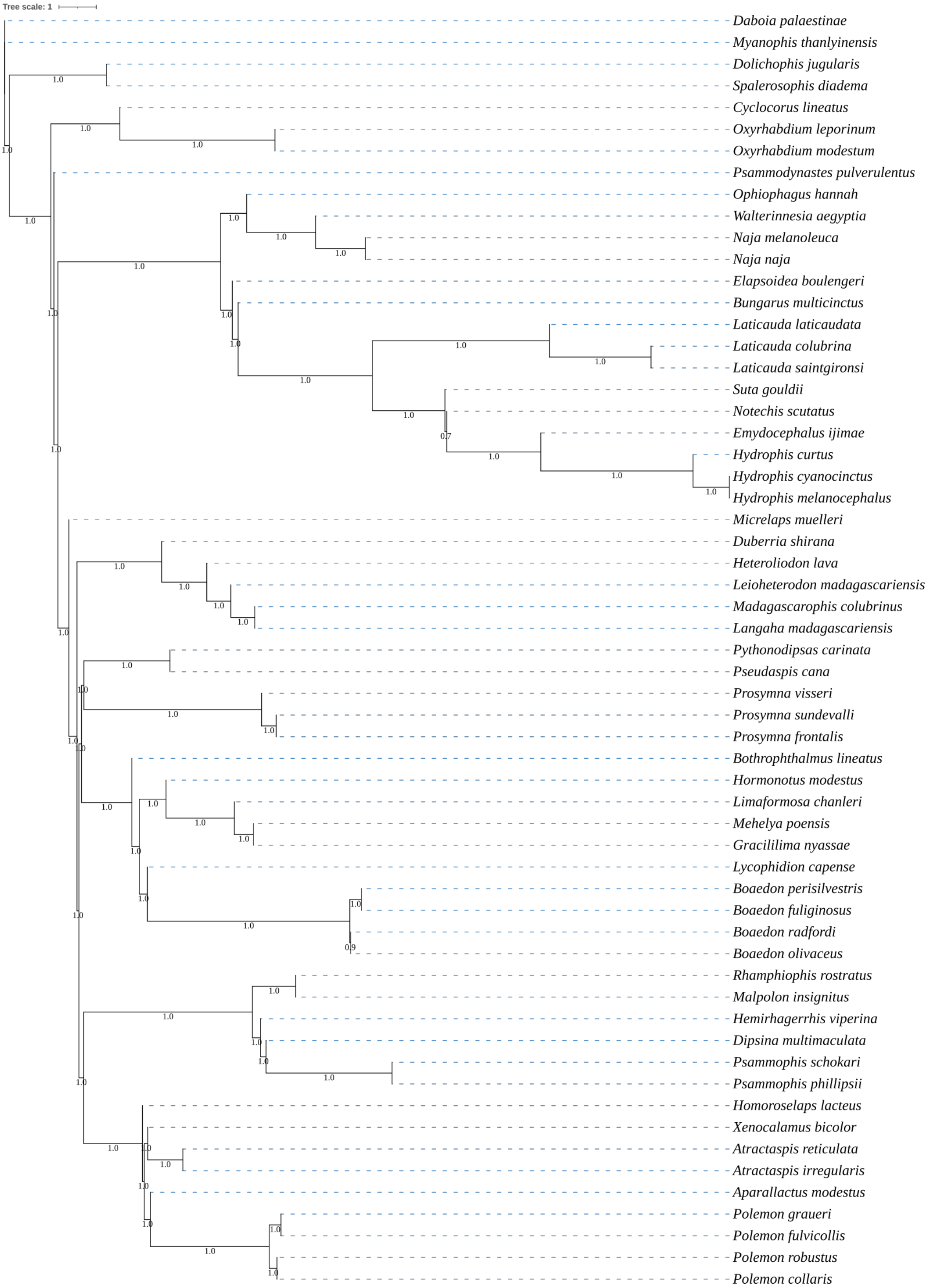

Fig. S1: 50 % complete UCE wASTRAL-h phylogeny. LocalPP on the branches.

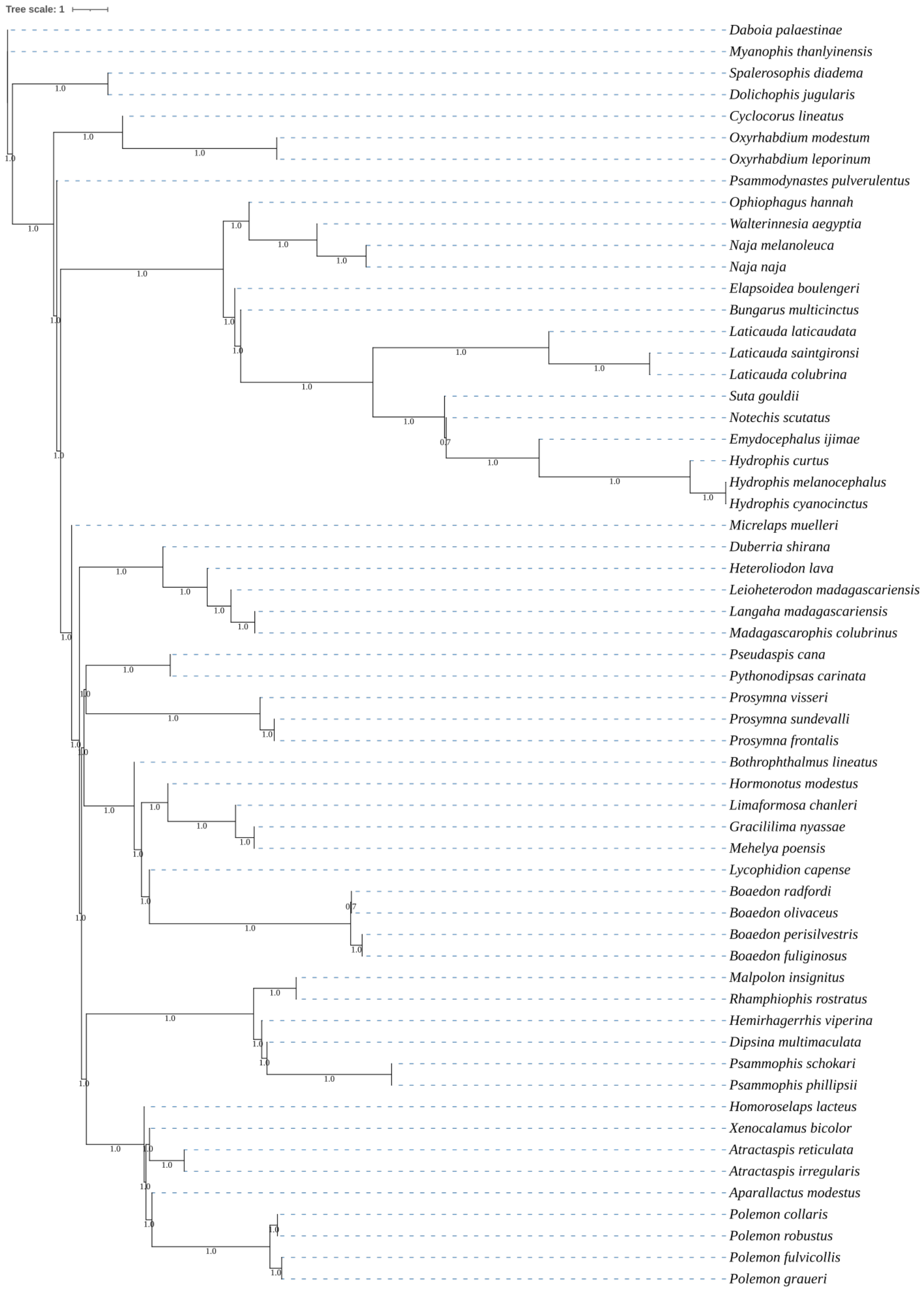

Fig. S2: 75 % complete UCE wASTRAL-h phylogeny. LocalPP on the branches.

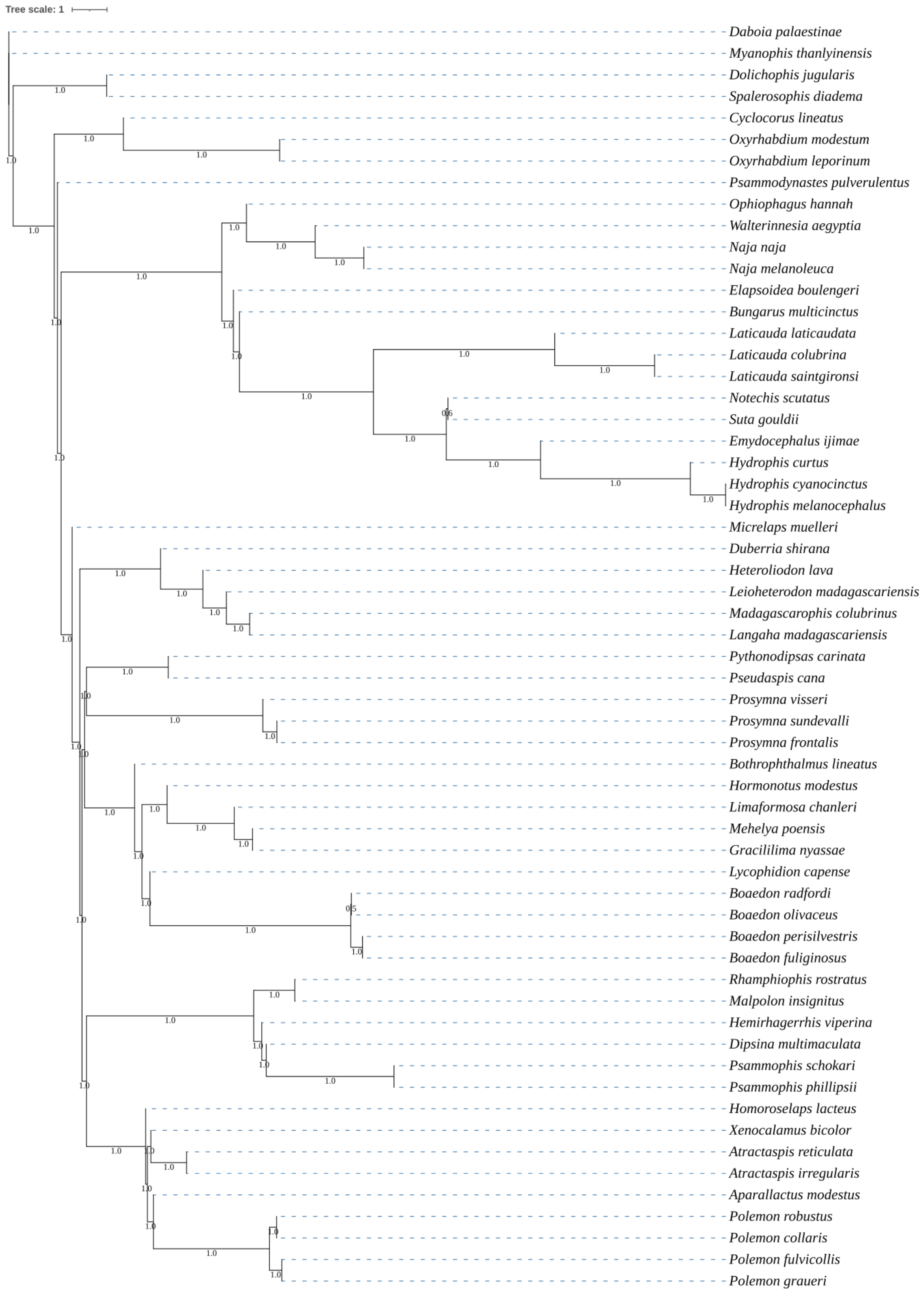

Fig. S3: 95 % complete UCE wASTRAL-h phylogeny. LocalPP on the branches.

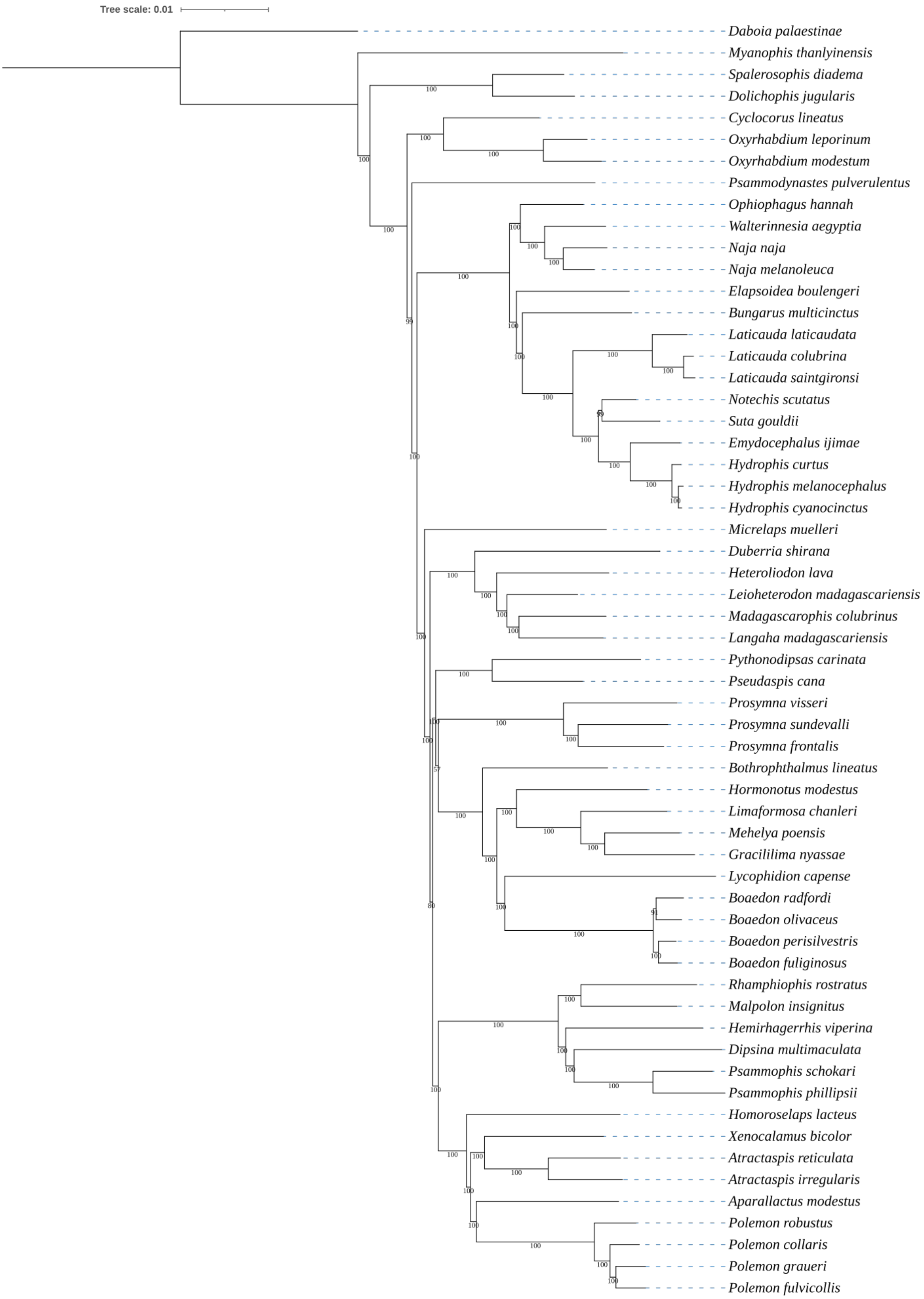

Fig. S4: 50 % complete UCE ML phylogeny. UFBoot on the branches.

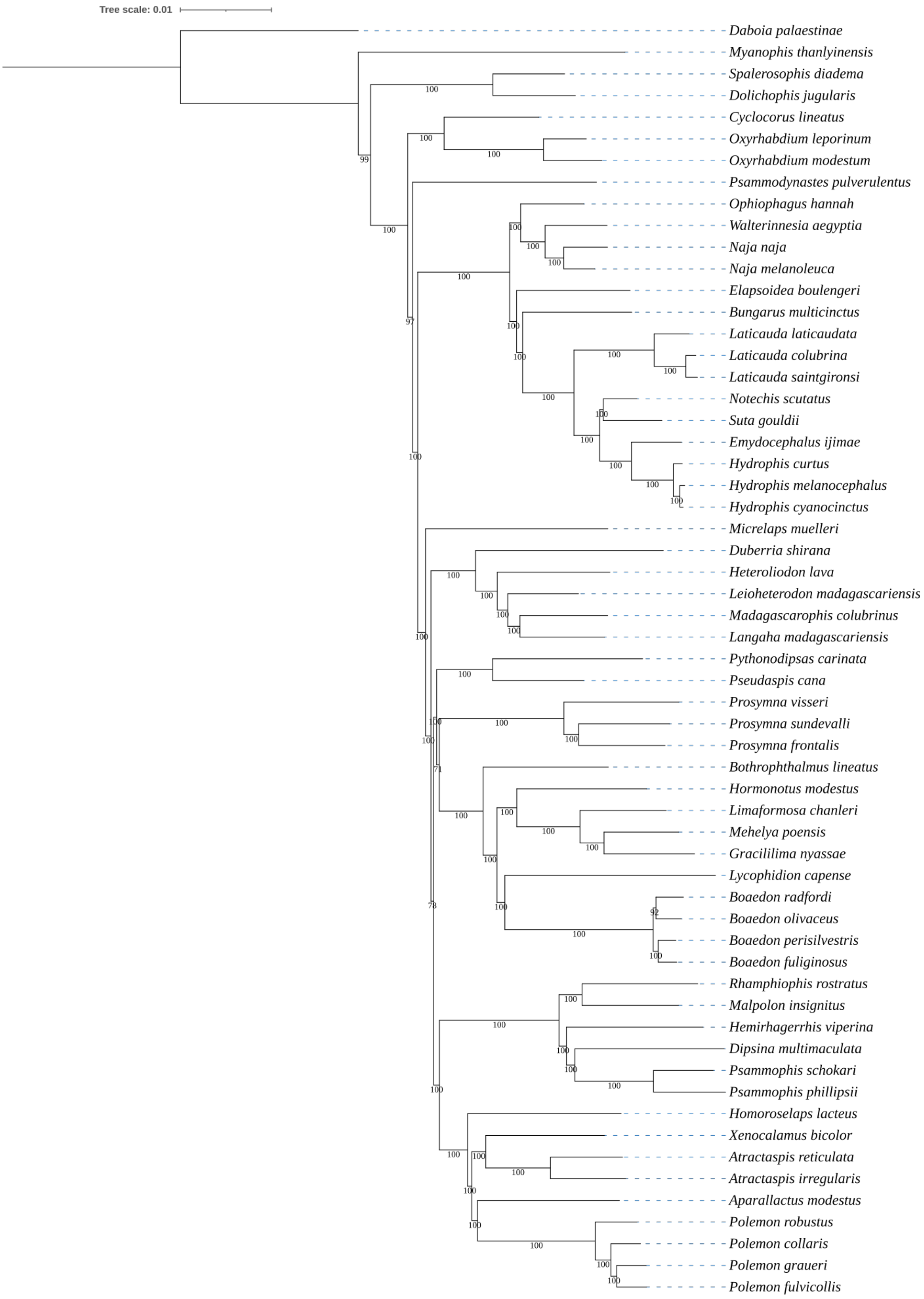

Fig. S5: 75 % complete UCE ML phylogeny. UFBoot on the branches.

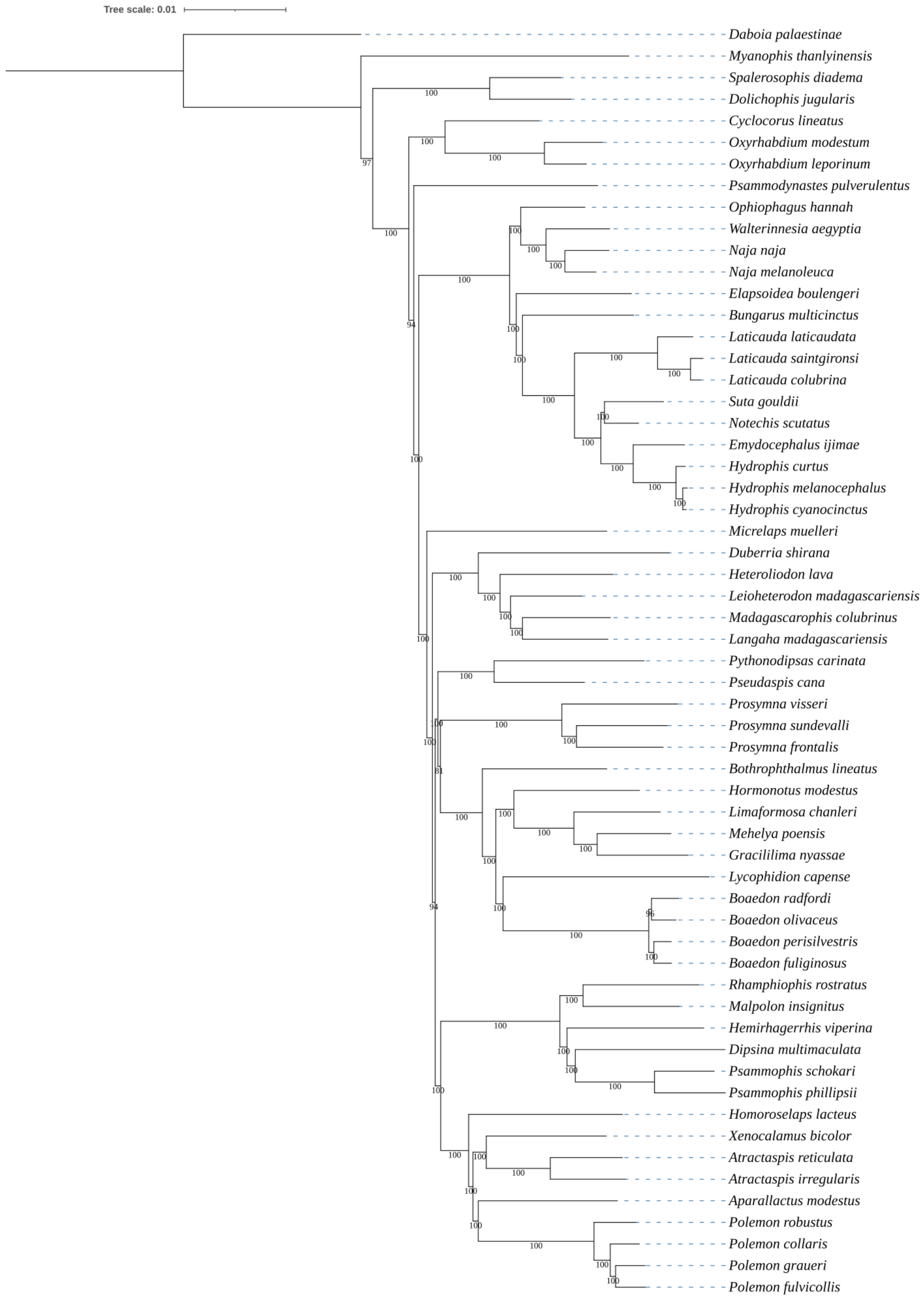

Fig. S6: 95 % complete UCE ML phylogeny. UFBoot on the branches.

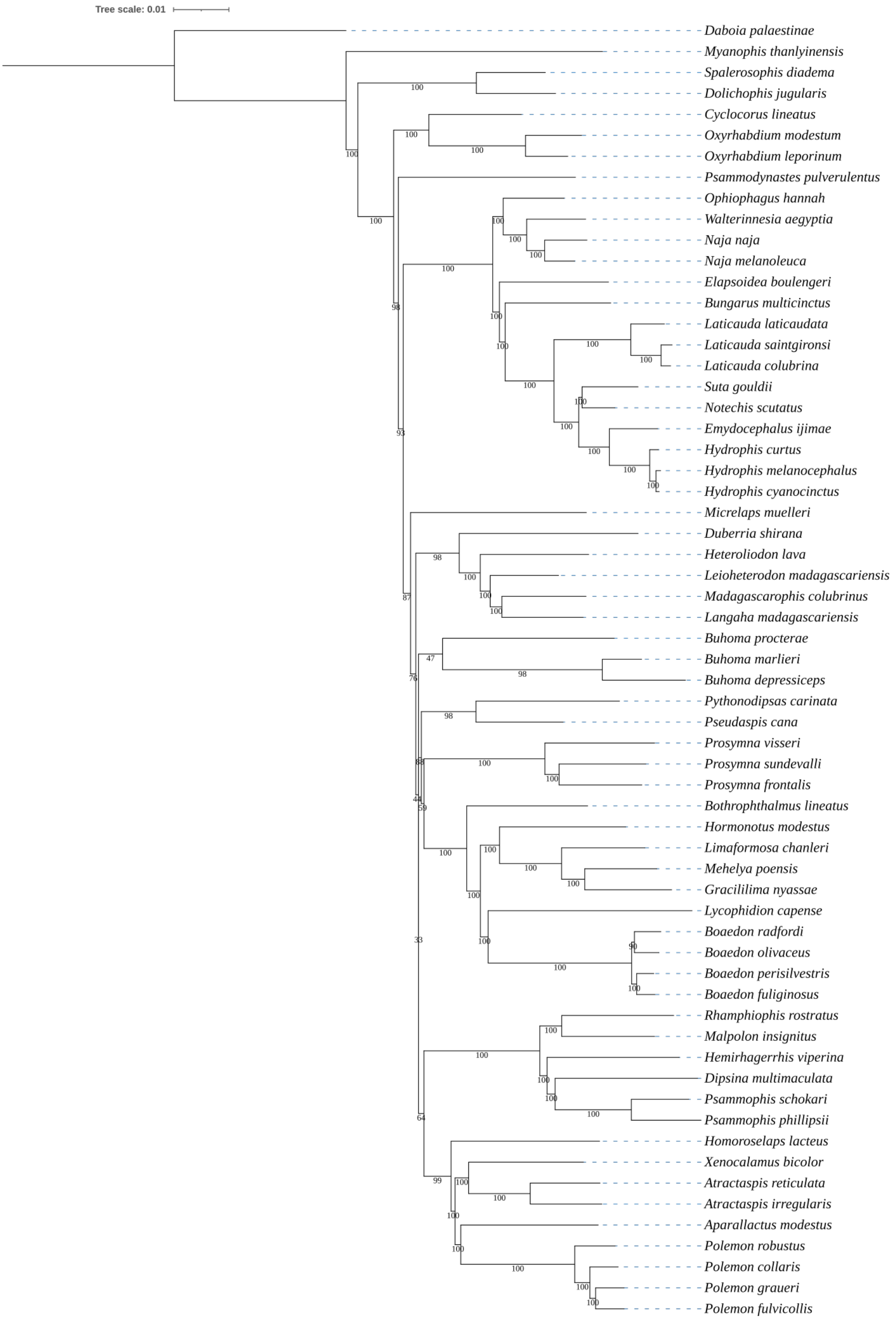

Fig. S7: 50 % UCE + tradition nuclear marker ML phylogeny. UFBoot on branches. For composite taxa (named here as in UCE datatset), see Figure 2 and Table S1.

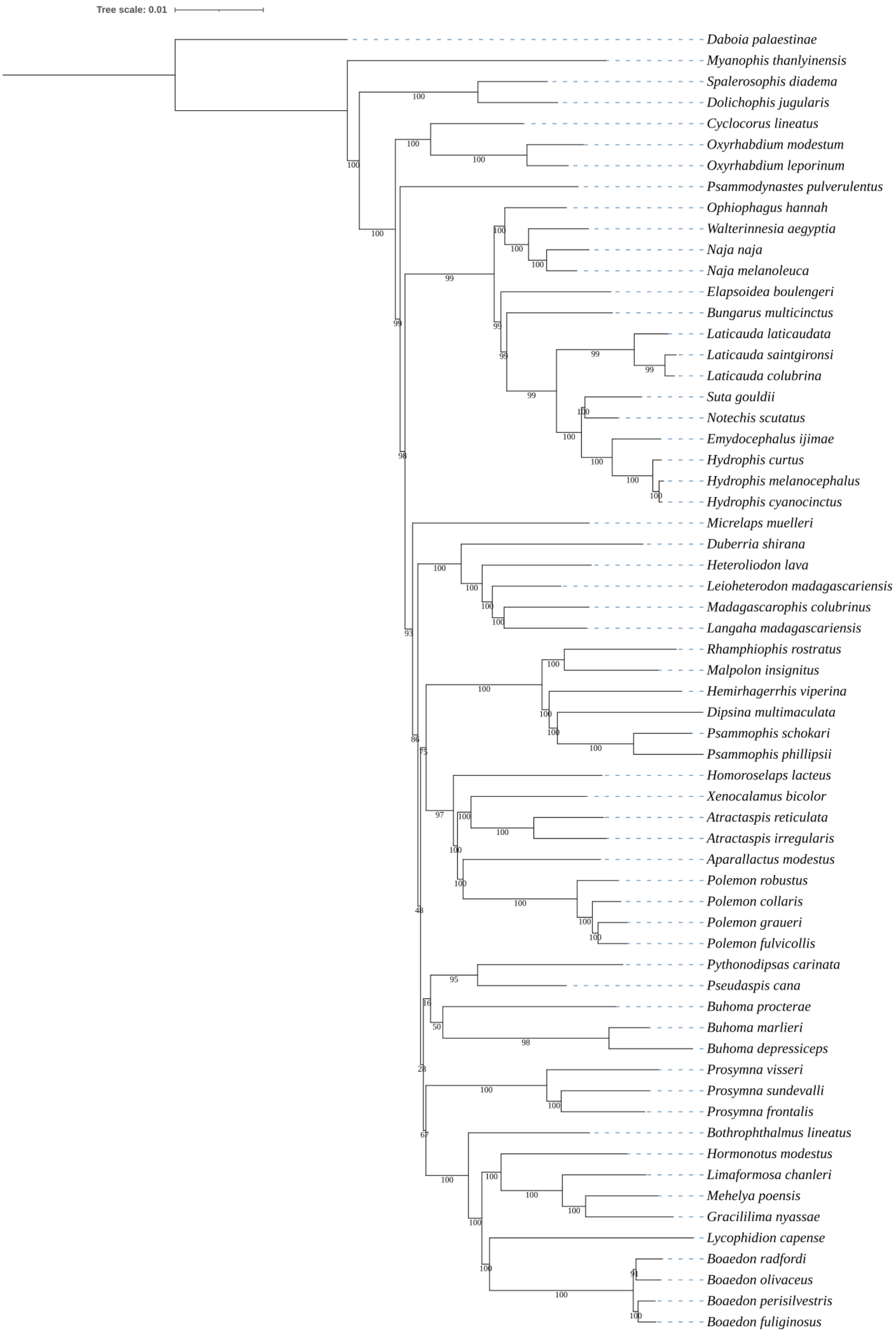

Fig. S8: 75 % UCE + tradition nuclear marker ML phylogeny. UFBoot on branches. For composite taxa (named here as in UCE datatset), see Figure 2 and Table S1.

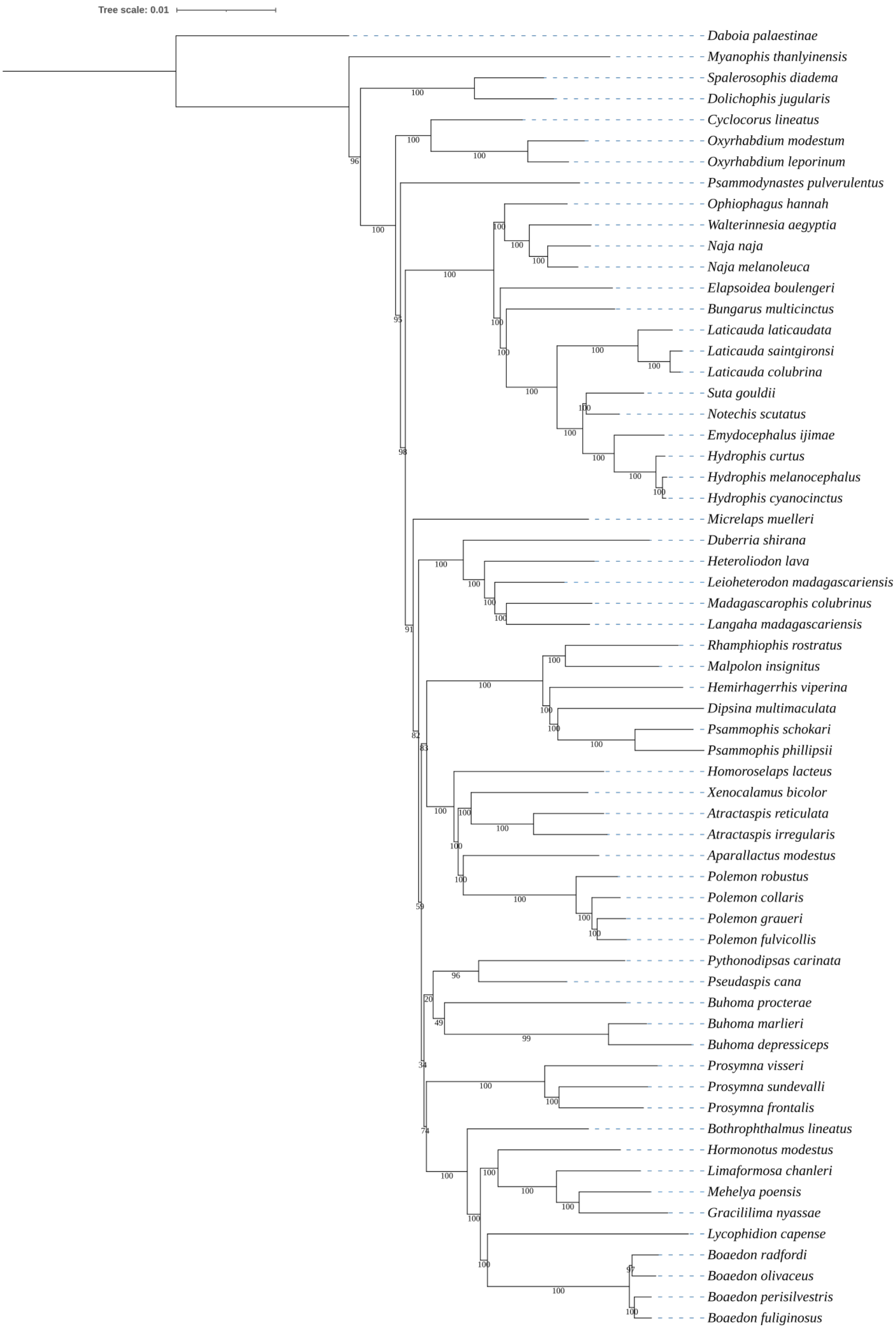

Fig. S9: 95 % UCE + tradition nuclear marker ML phylogeny. UFBoot on branches. For composite taxa (named here as in UCE datatset), see Figure 2 and Table S1.

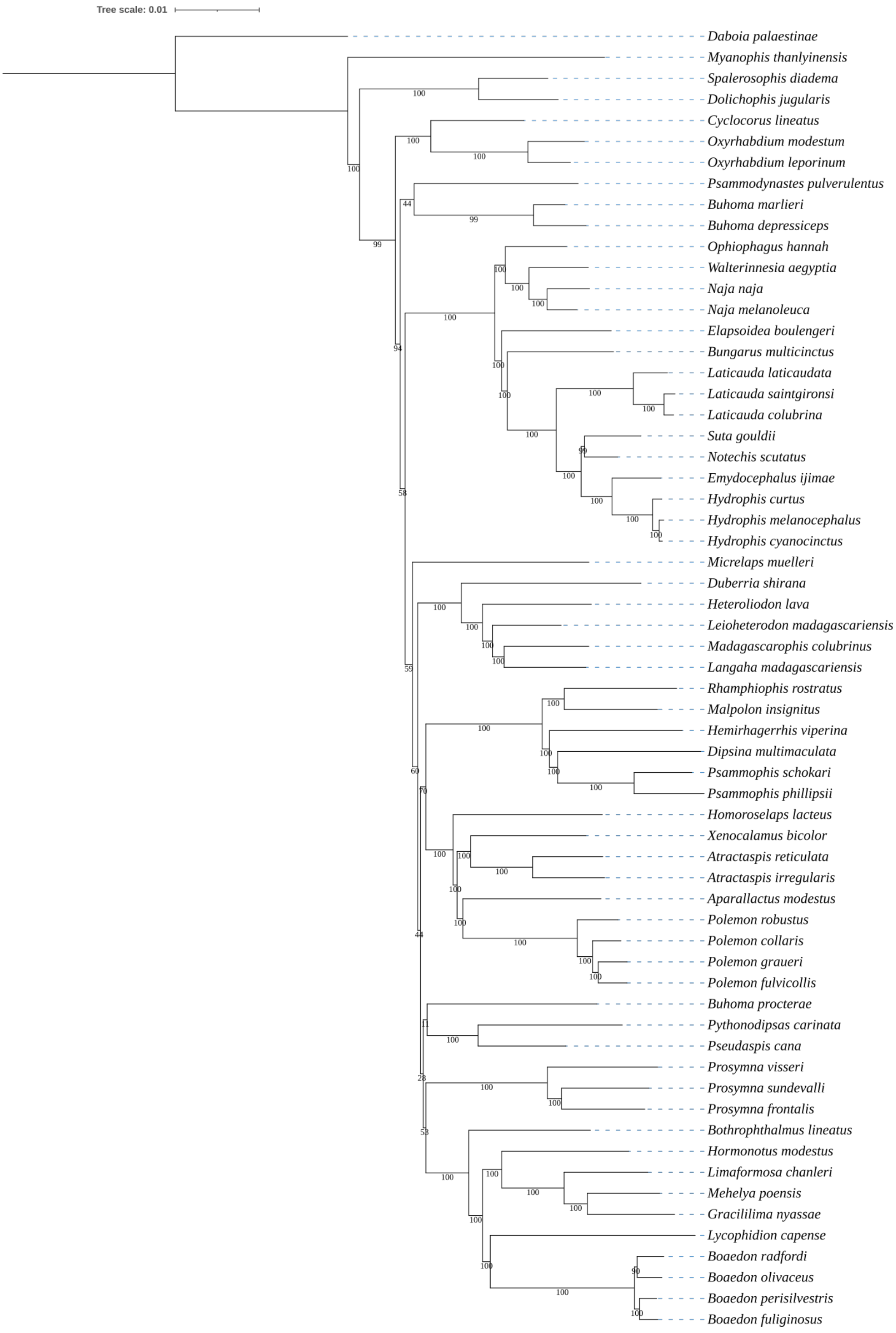

Fig. S10: 50 % UCE + tradition mito-nuclear marker ML phylogeny. UFBoot on branches.

For composite taxa (named here as in UCE datatset), see Table S1.

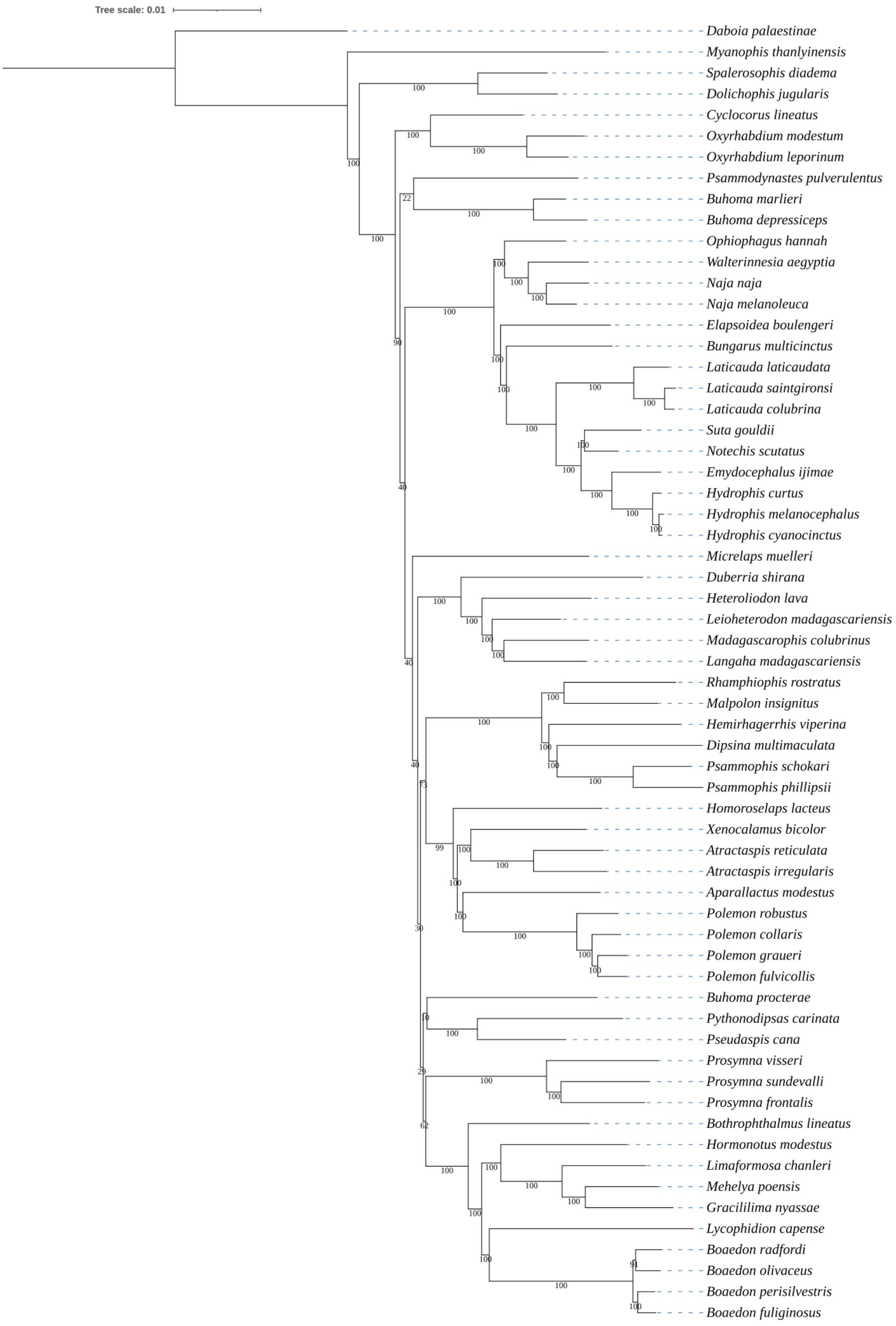

Fig. S11: 75 % UCE + tradition mito-nuclear marker ML phylogeny. UFBoot on branches.

For composite taxa (named here as in UCE datatset), see Table S1.

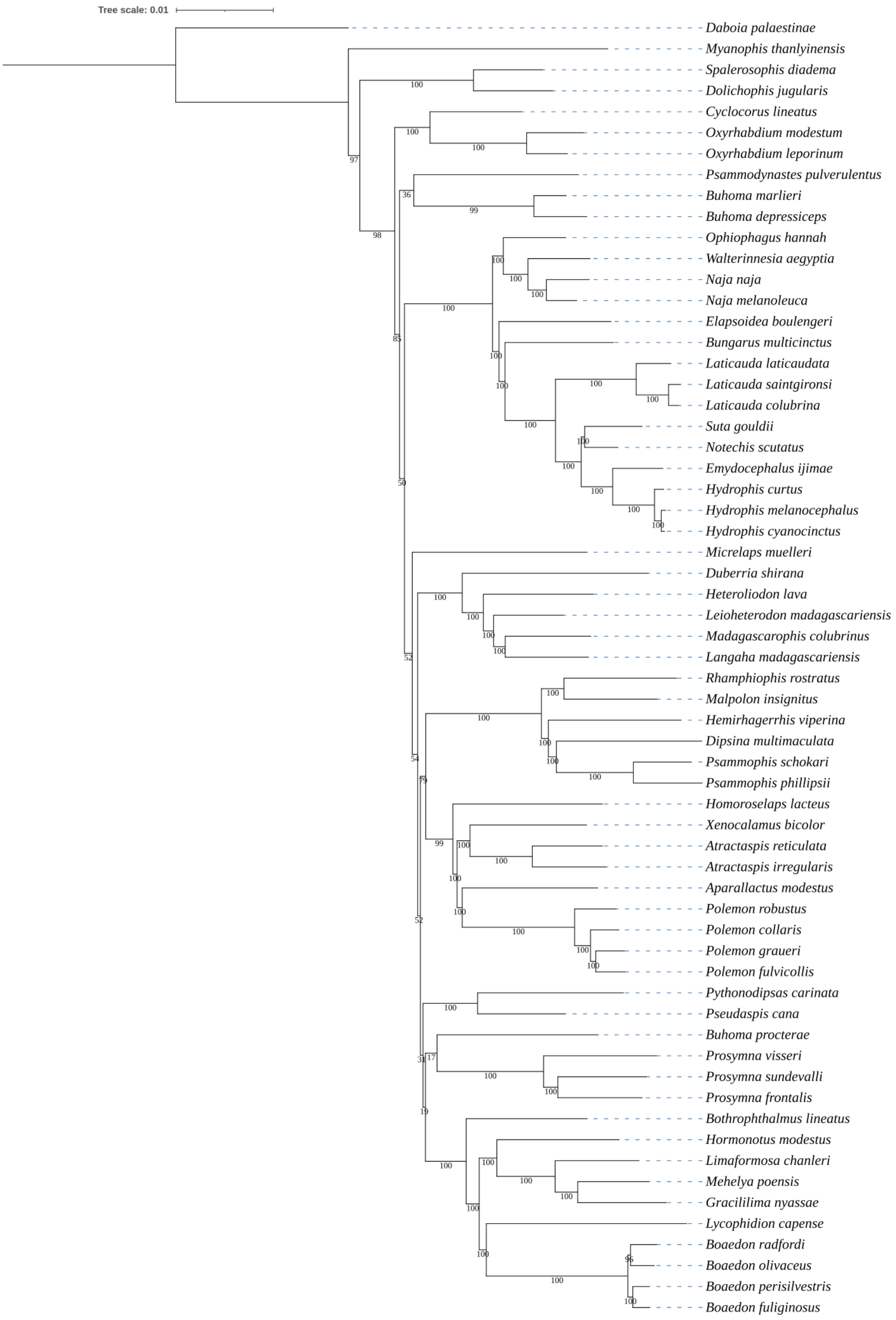

Fig. S12: 95 % UCE + tradition mito-nuclear marker ML phylogeny. UFBoot on branches.

For composite taxa (named here as in UCE datatset), see Table S1.

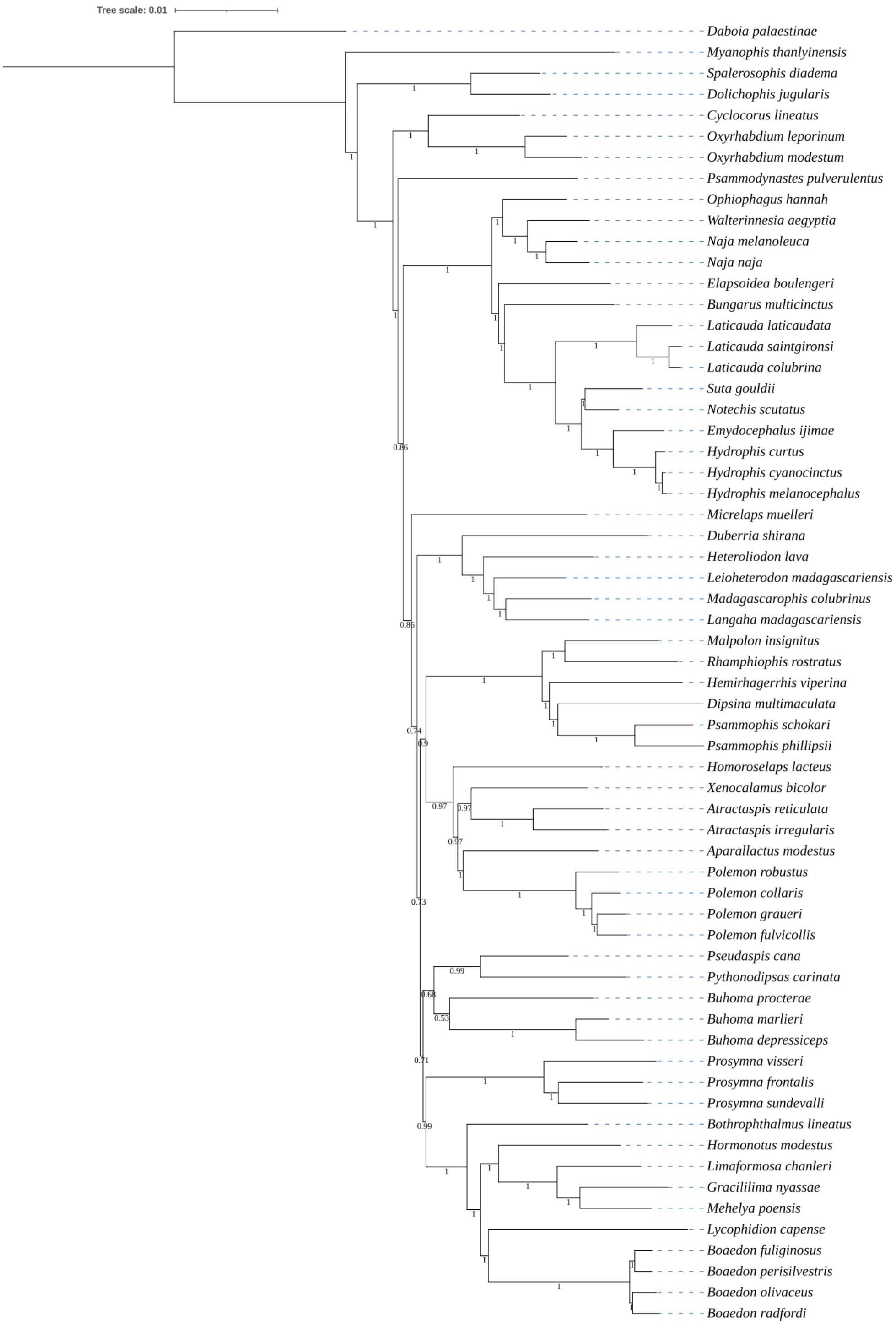

Fig. S13: 95 % UCE and traditional nuclear marker ExaBayes phylogeny. Posterior probability on the branches. For composite taxa (named here as in UCE datatset), see Figure 2 and Table S1.

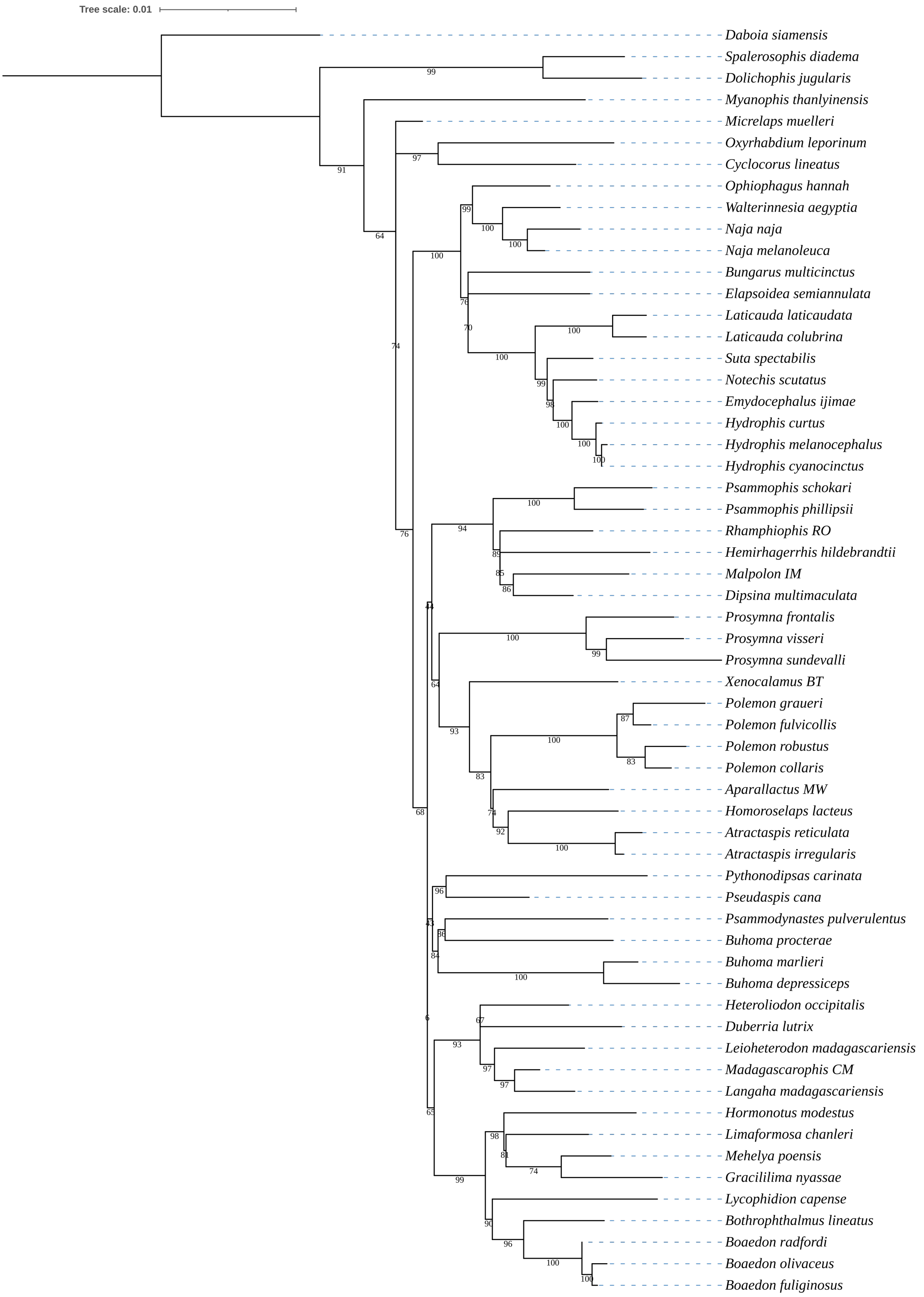

Fig. S14: Traditional nuclear marker ML phylogeny. UFBoot on the branches. For composite taxa, see Table S1.

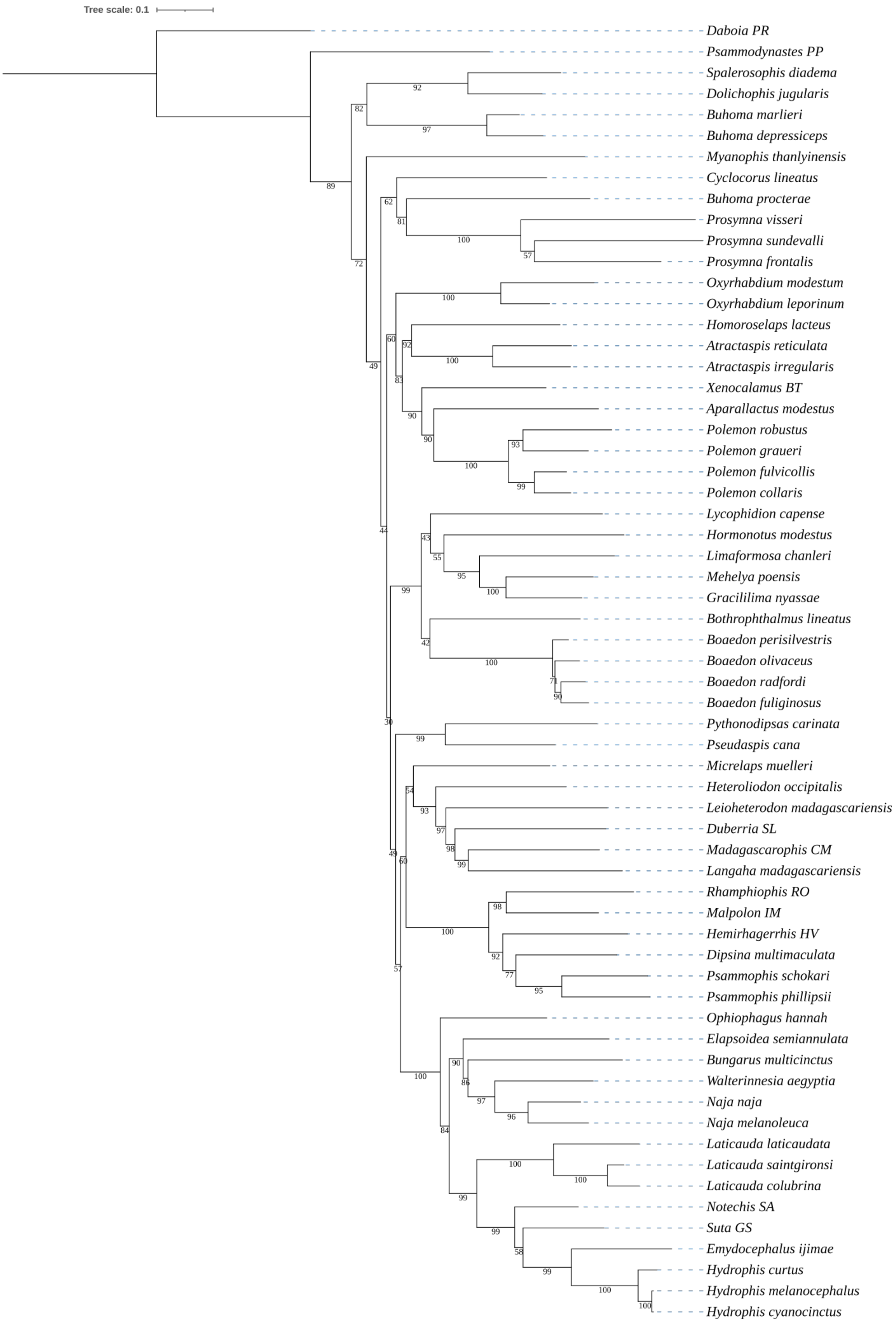

Fig. S15: Traditional mitochondrial marker ML phylogeny. UFBoot on the branches. For composite taxa (the ones with capital letters in the place of the specific epithet), see Table S1.

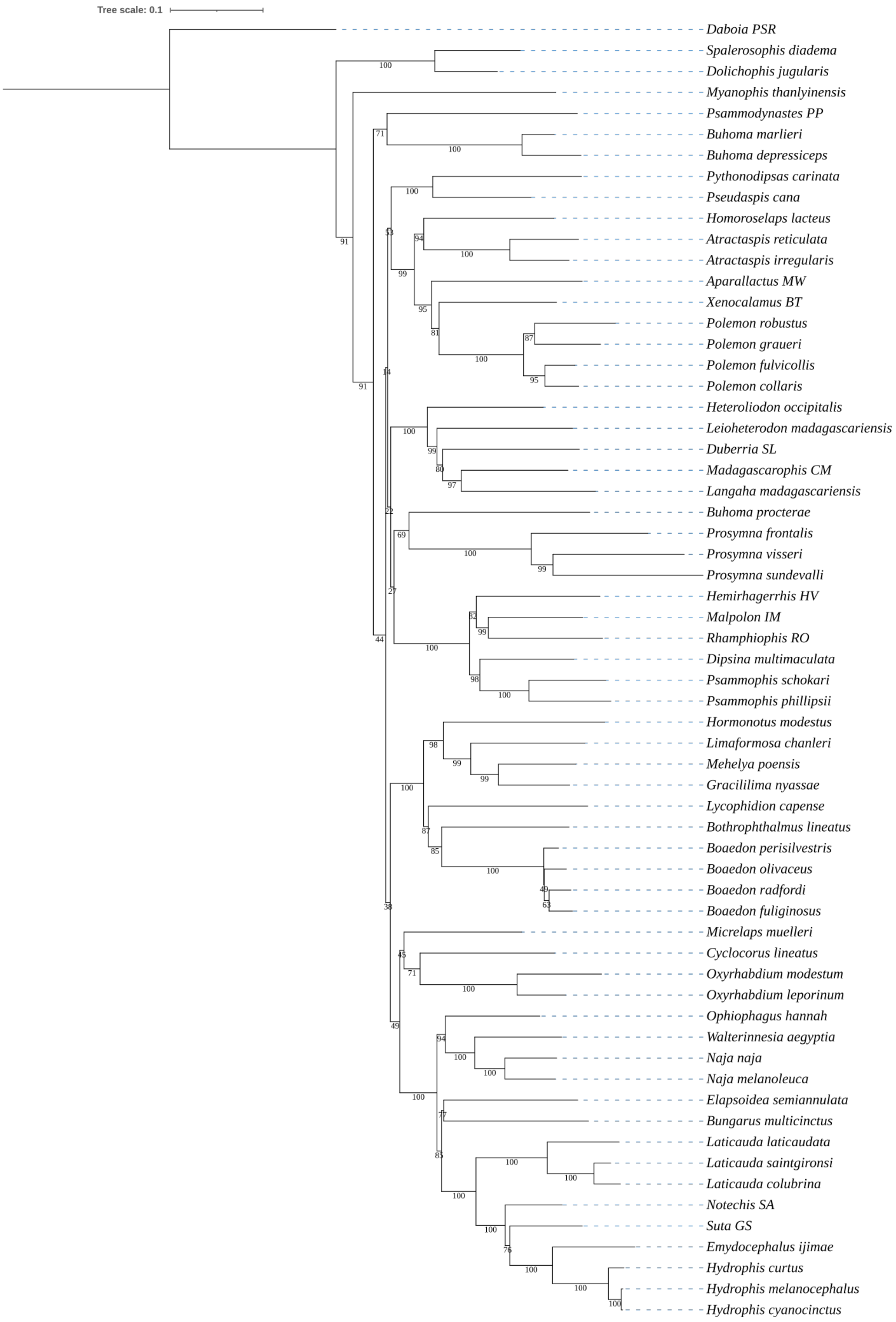

Fig. S16: Traditional mito-nuclear marker ML phylogeny. UFBoot on the branches. For composite taxa (the ones with capital letters in the place of the specific epithet), see Table S1.

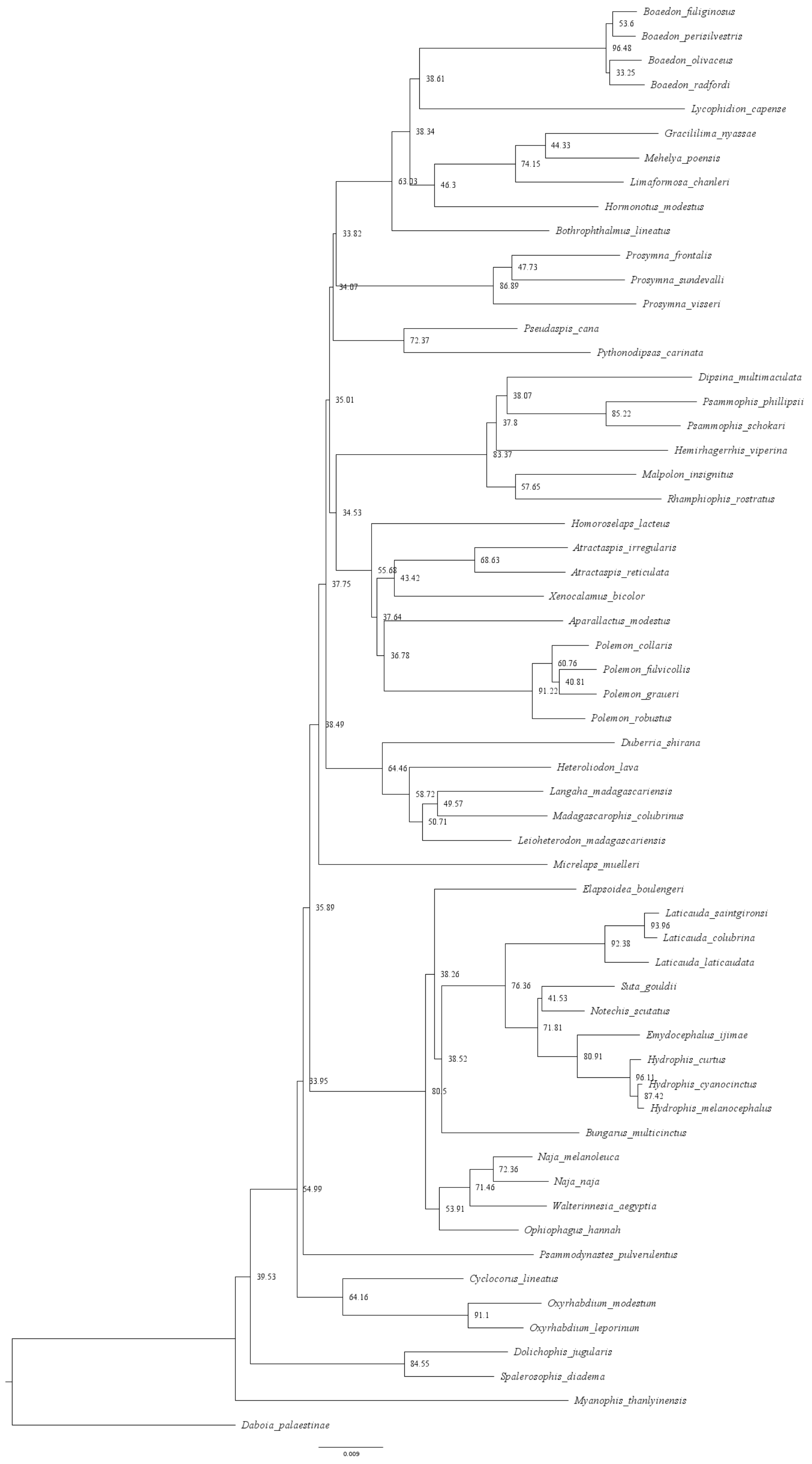

Fig. S17: Site concordance factors received by the branches in the 50 % complete UCE ML phylogeny.

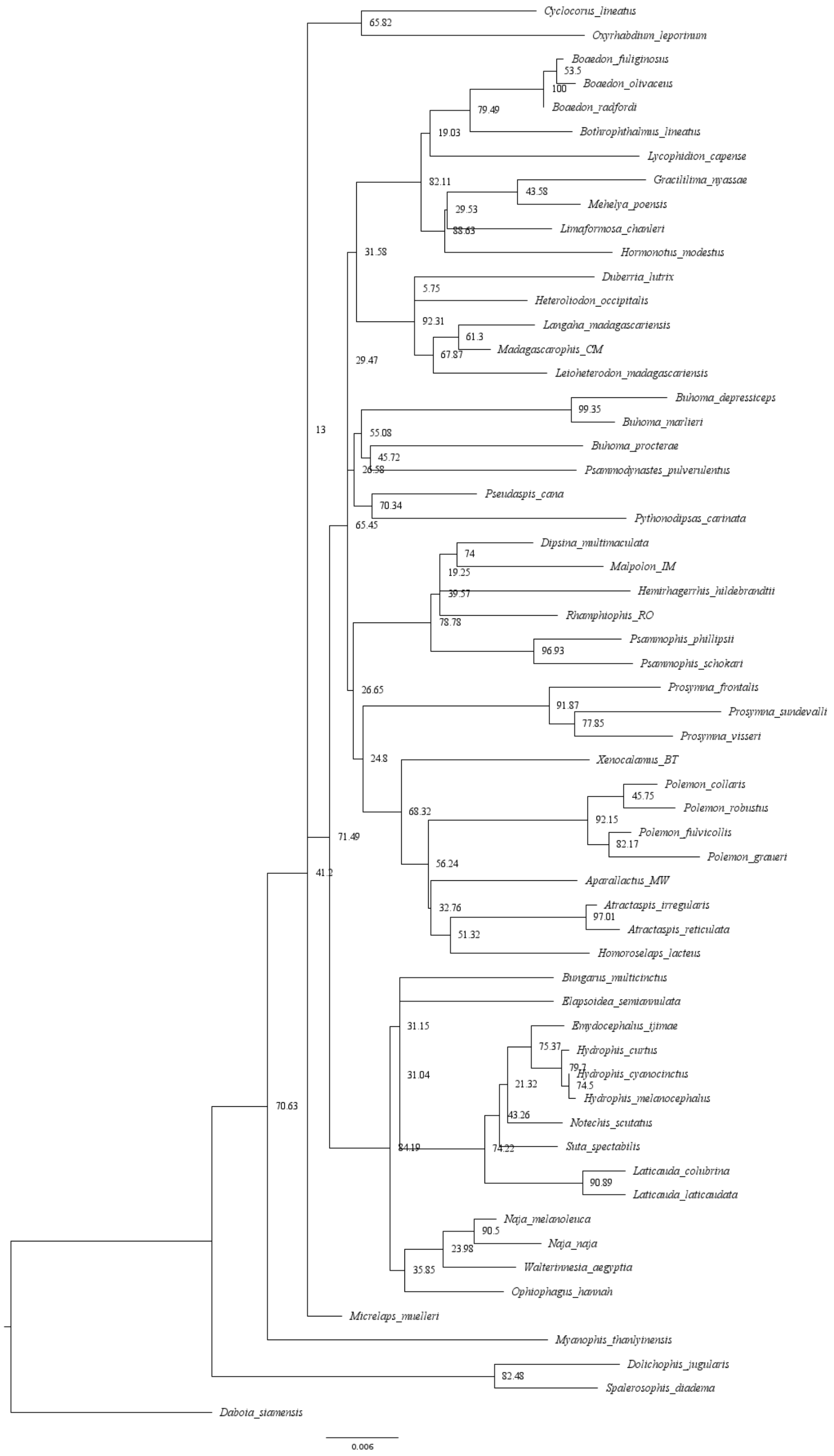

Fig. S18: Site concordance factors received by the branches in the traditional nuclear marker ML phylogeny.

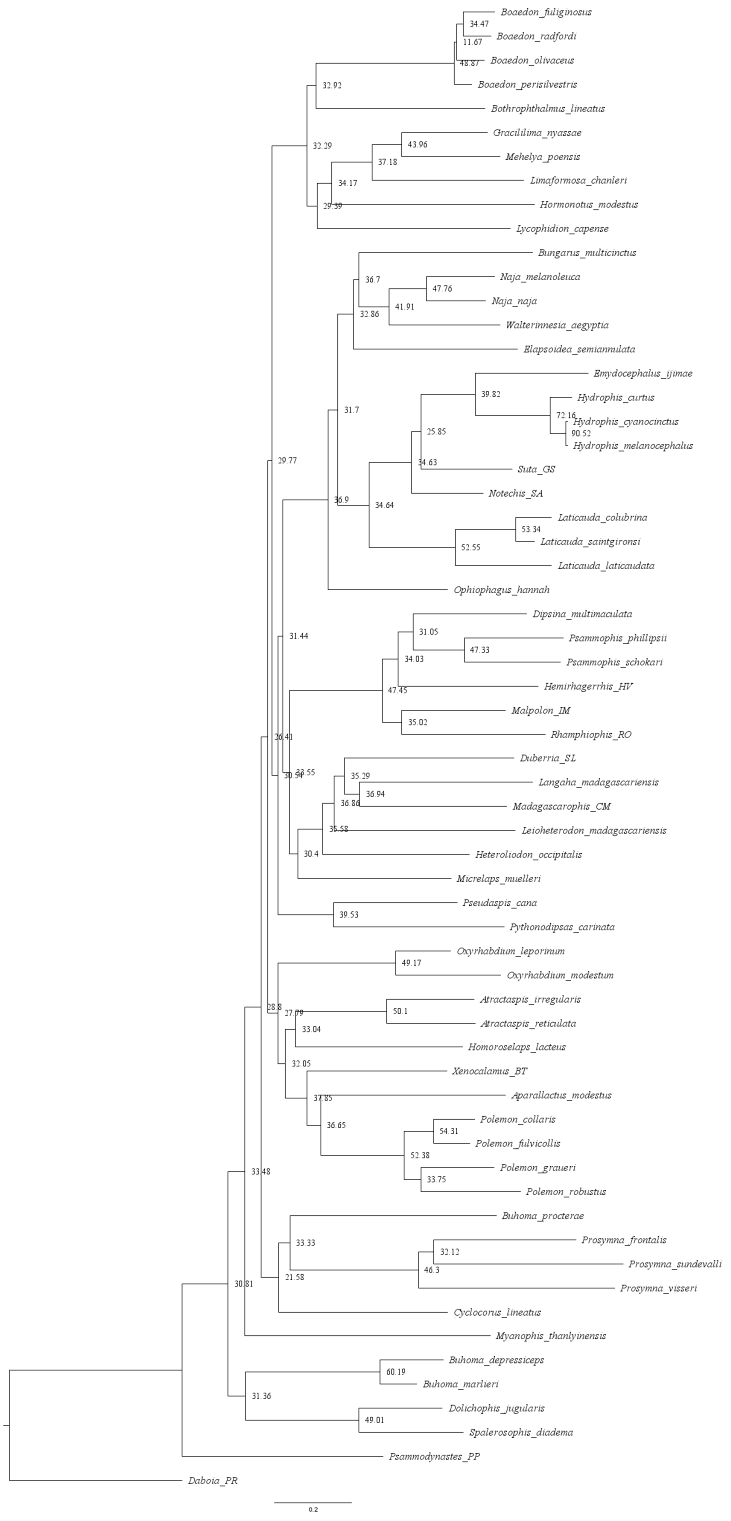

Fig. S19: Site concordance factors received by the branches in the traditional mitochondrial marker ML phylogeny.

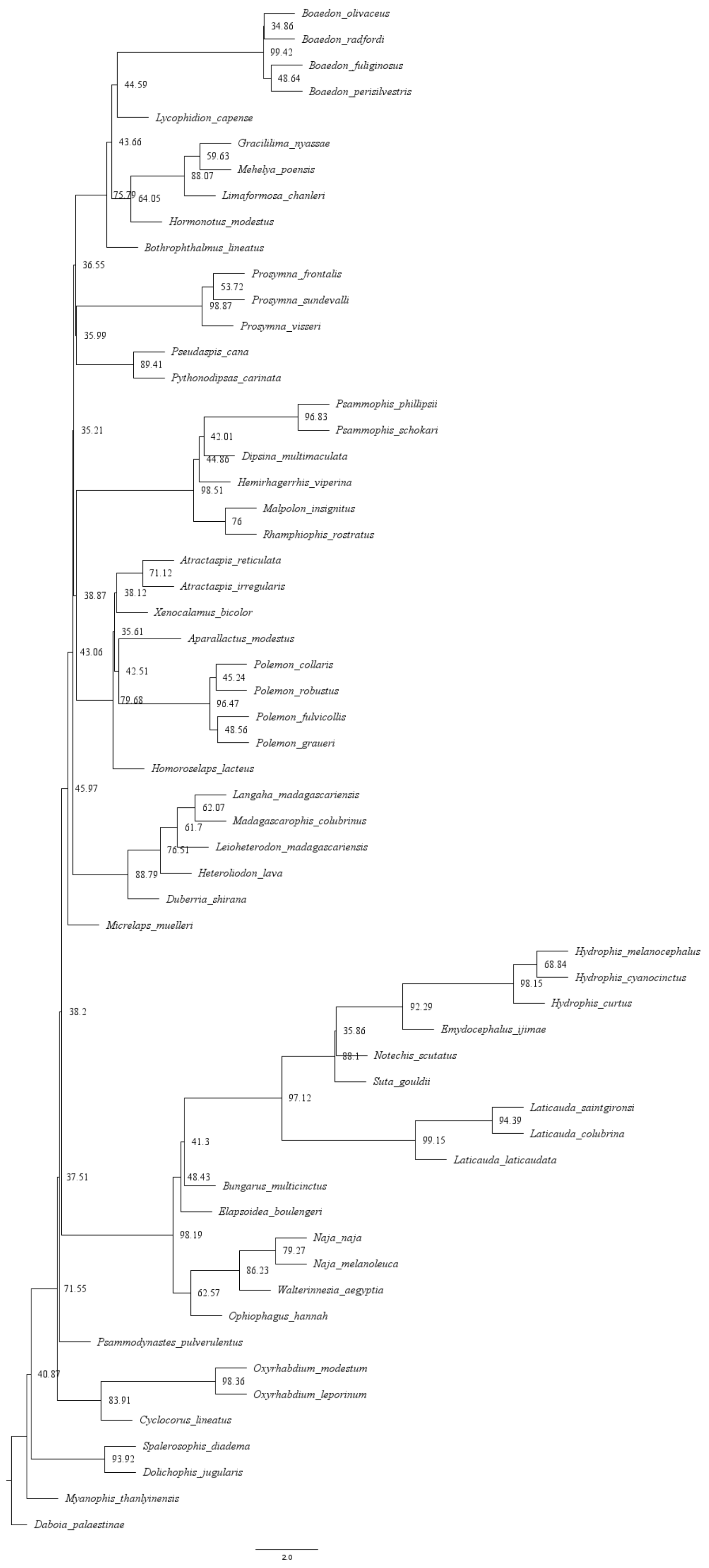

Fig. S20: Quartet support on the branches of the 50 % complete UCE wASTRAL-h phylogeny.

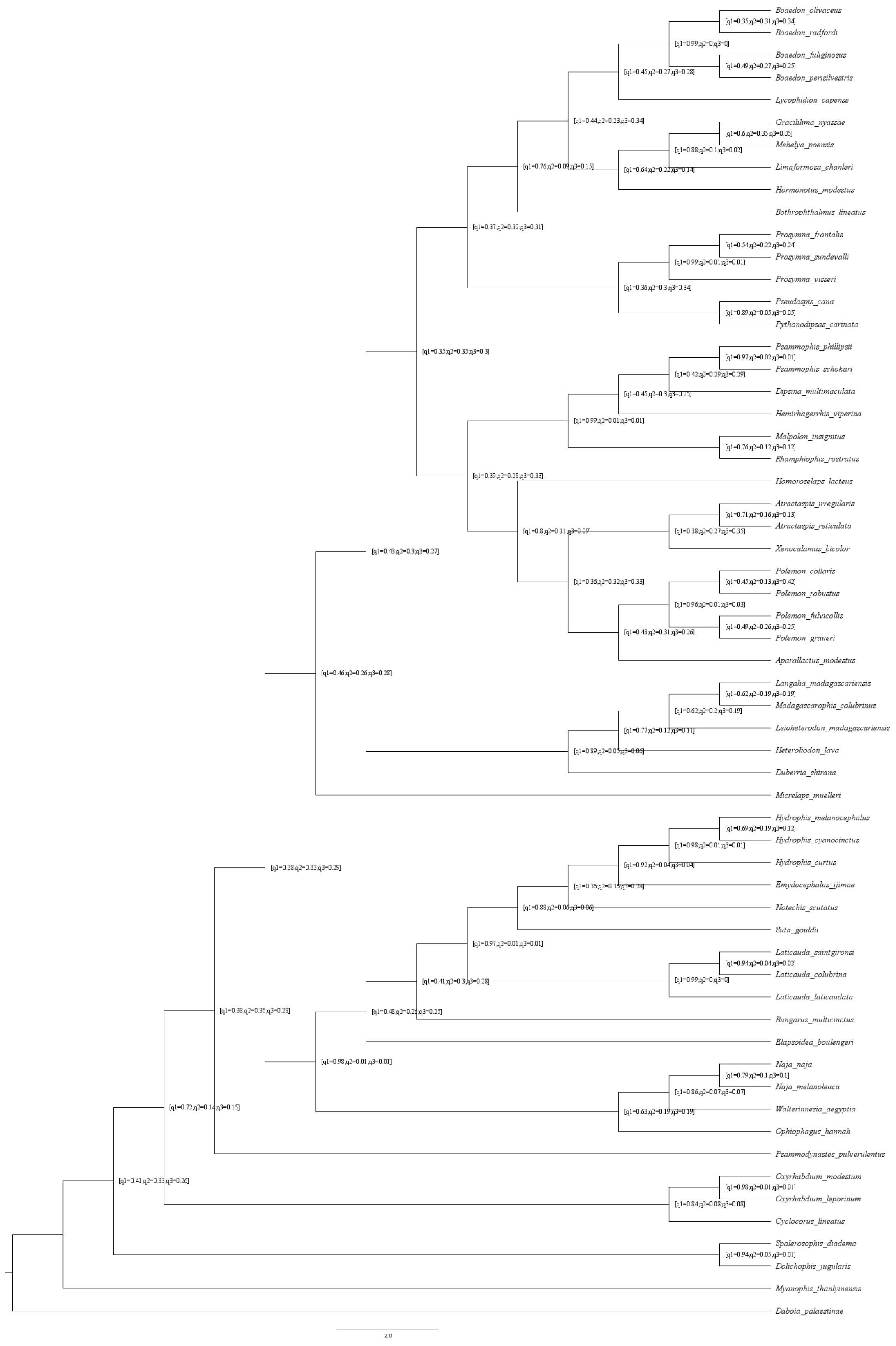

Fig. S21: Quartet support for the best and the alternative resolutions of the 50 % complete UCE wASTRAL-h phylogeny.

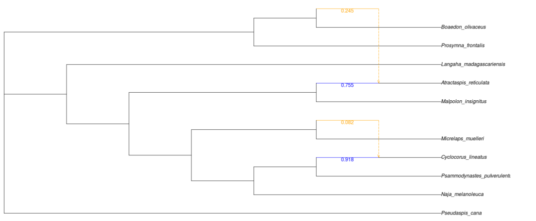

Allowed reticulations (hmax) = 2

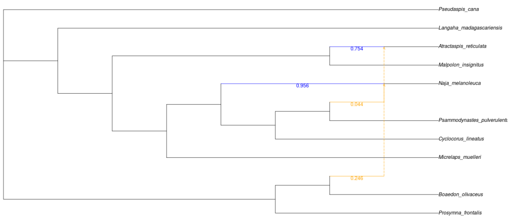

Allowed reticulations (hmax) = 5

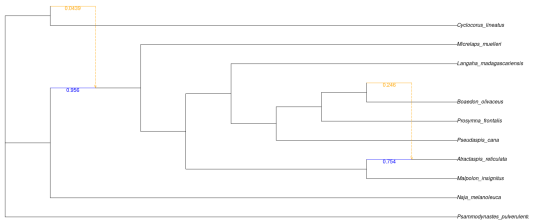

Allowed reticulations (hmax) = 6

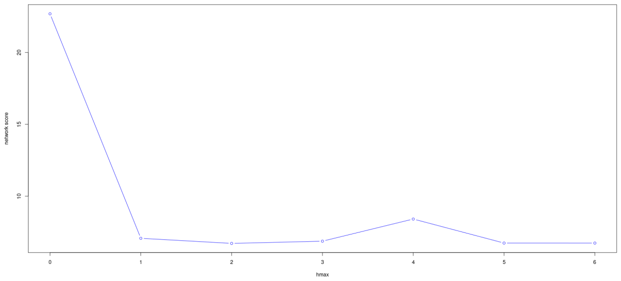

Network score (log-likelihoods treated as scores) plotted again hmax

Fig. S22: Three top log-likelihood achieving MSCNs estimated by PhyloNetworks and plot of reticulations again the log-likelihoods in the visualisation format preferred by PhyloNetworks developers.

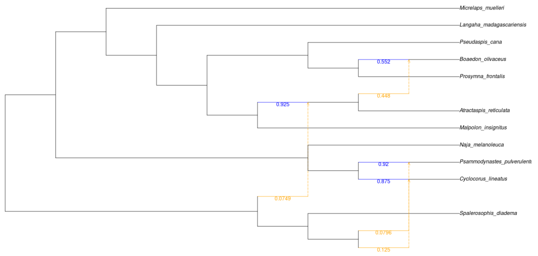

Allowed reticulations = 4

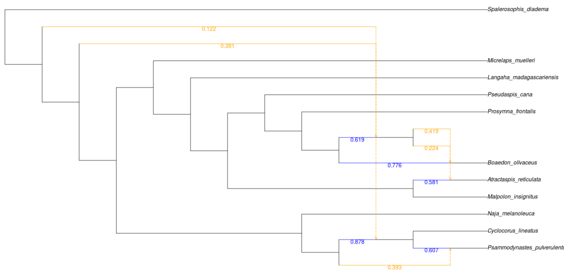

Allowed reticulations = 5

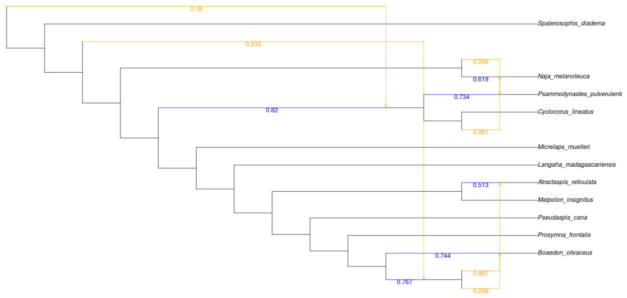

Allowed reticulations = 6

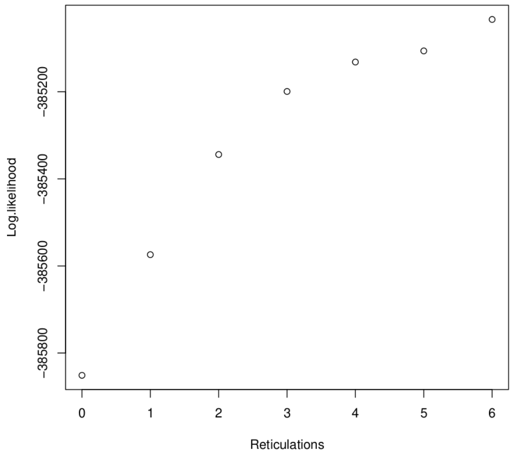

Log-likelihoods plotted against reticulations

Fig. S23: Three top log-likelihood achieving MSCNs estimated by PhyloNet and plot of reticulations again the log-likelihoods (the plot basically conveys the same kind of information as in the previous figure).

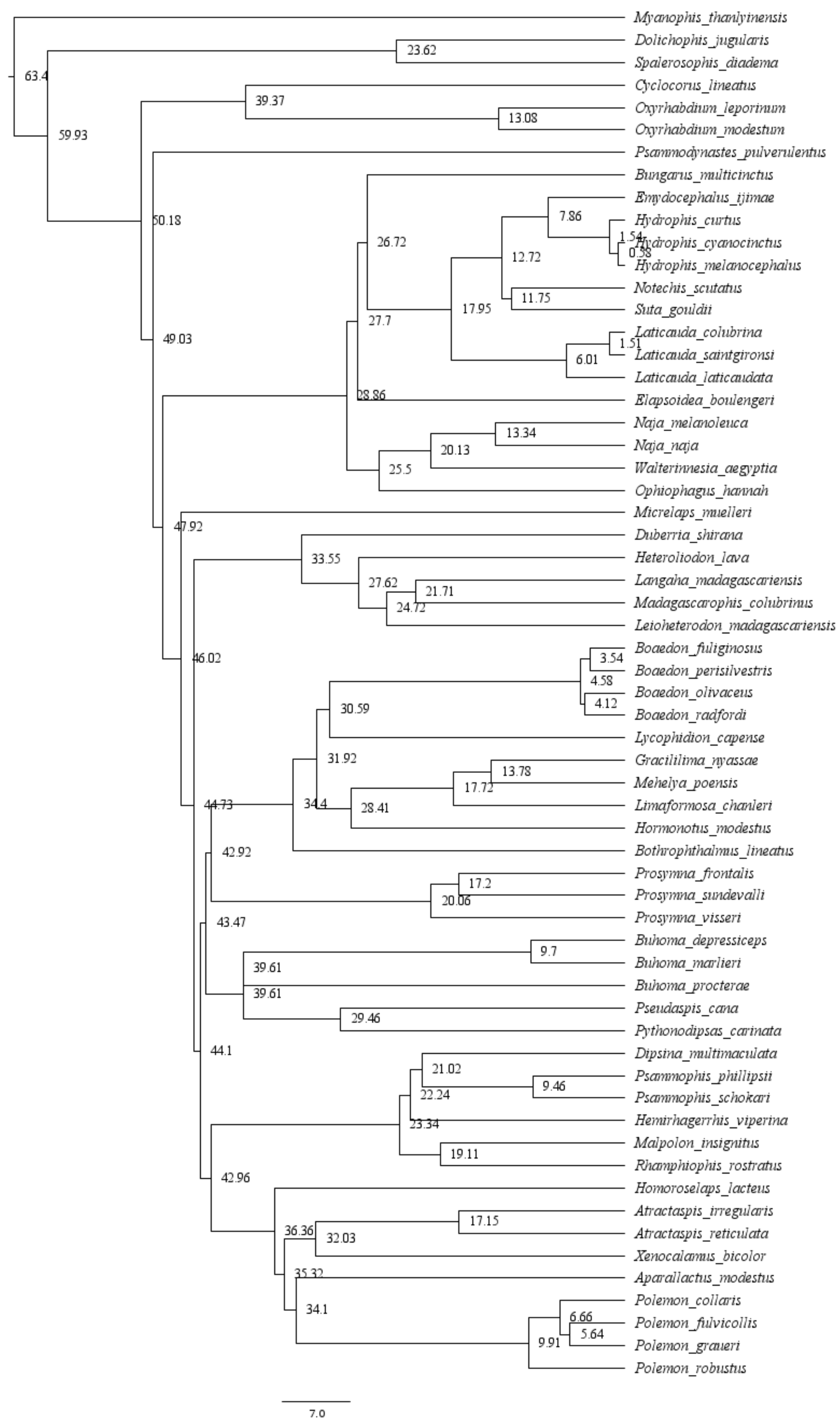

Fig. S24: Time-calibrated phylogeny of Elapoidea, from 95 % UCE and traditional nuclear loci dataset. Divergence times on the branches.

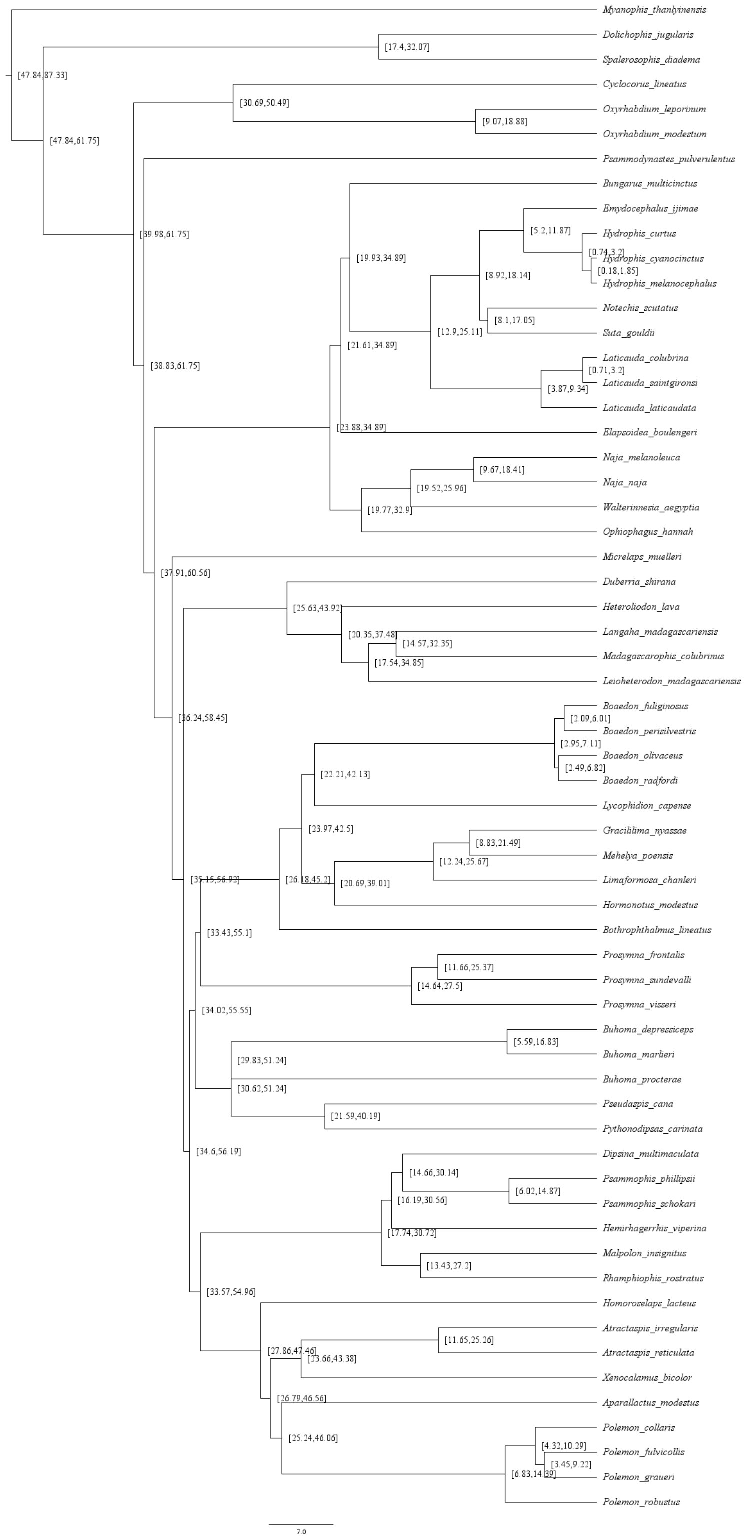

Fig. S25: Time-calibrated phylogeny of Elapoidea, from 95 % UCE and traditional nuclear loci dataset. 95 % confidence interval around the divergence times on the branches.

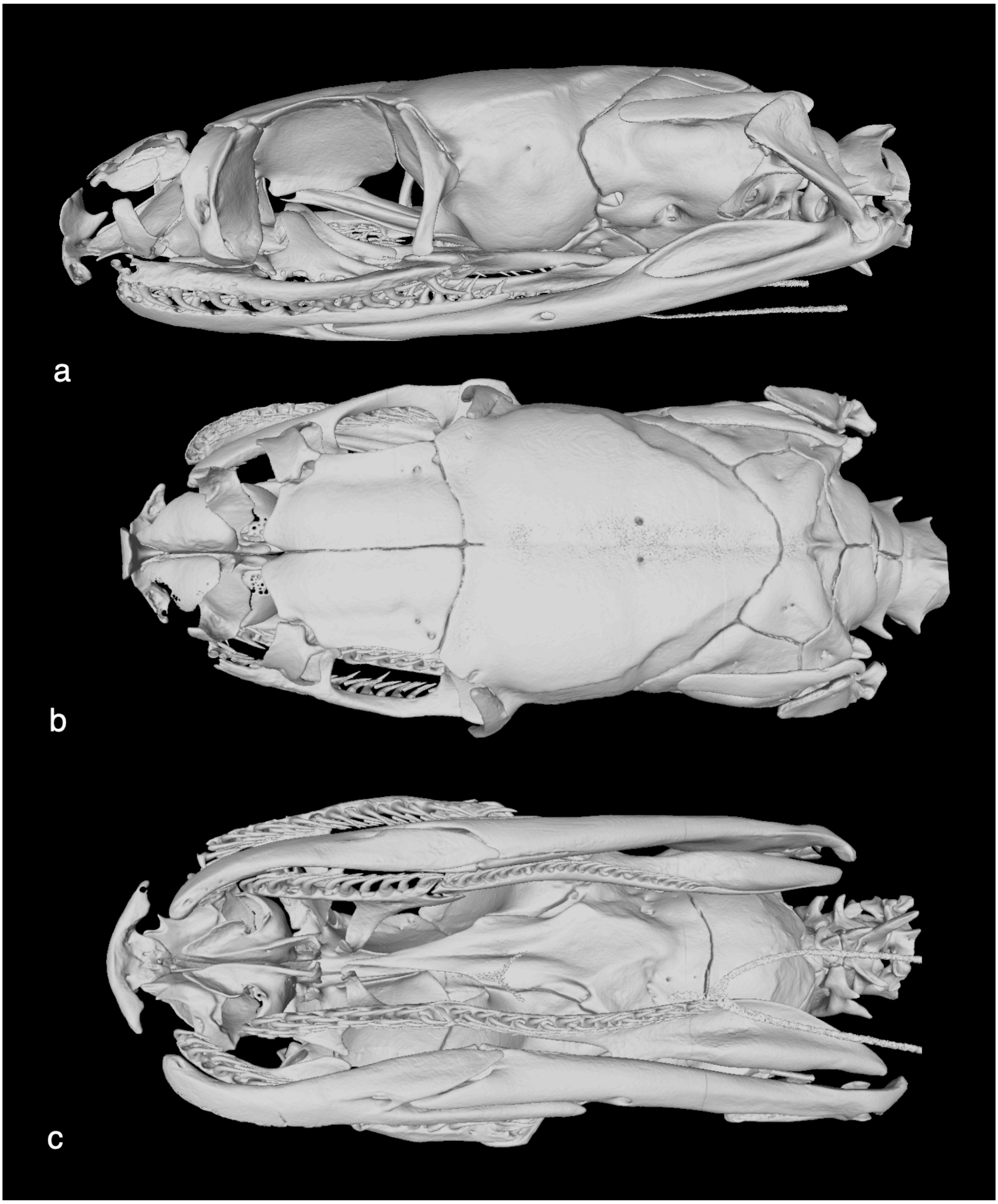

Fig. S26: Lateral (a), dorsal (b) and ventral (c) views of the cranium of *Buhoma marlieri* (BE-RBINS-VER-REP-8620)

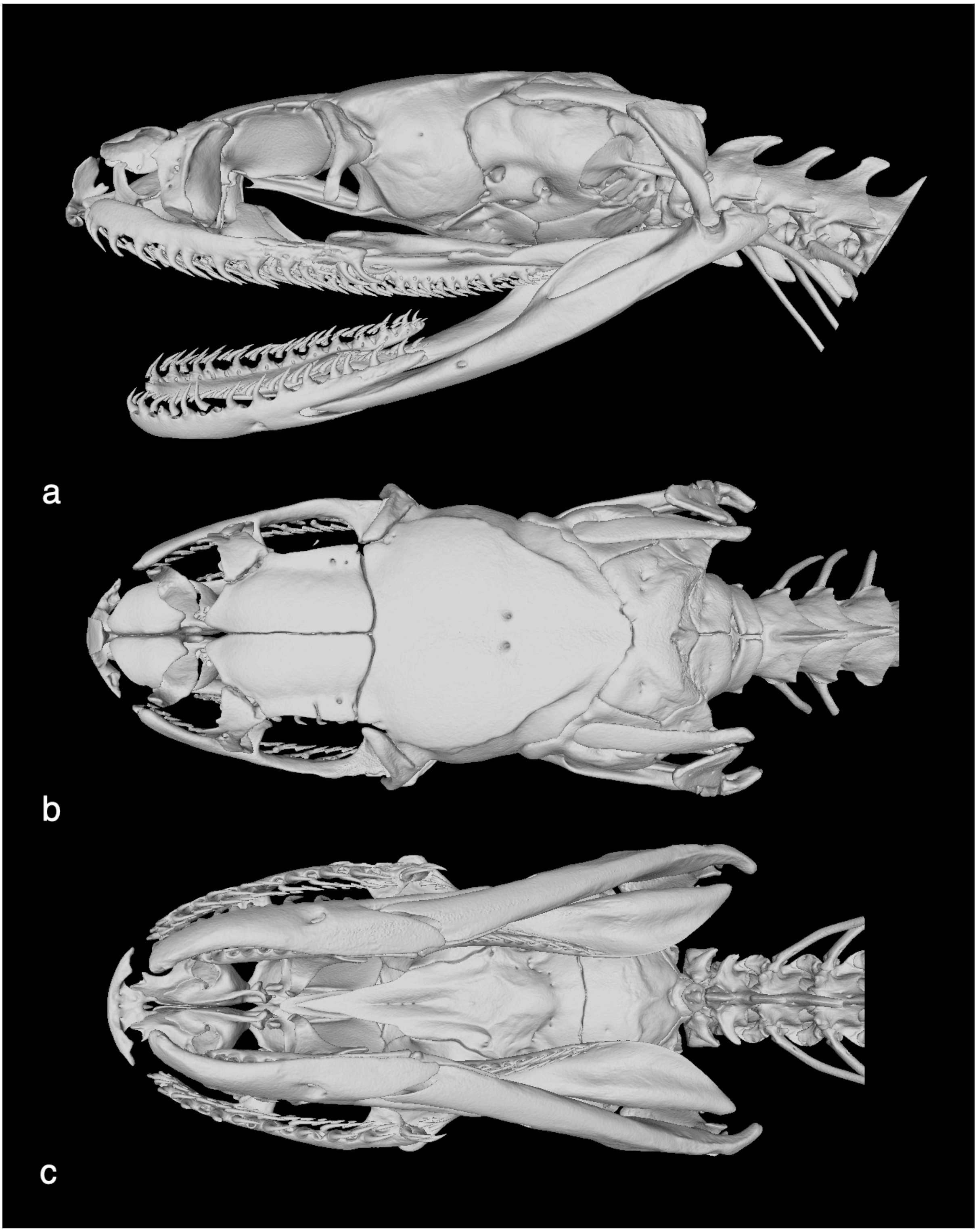

Fig. S27: Lateral (a), dorsal (b) and ventral (c) views of the cranium of the holotype of *Buhoma marlieri* (BE-RMCA-VER-REP 18091).

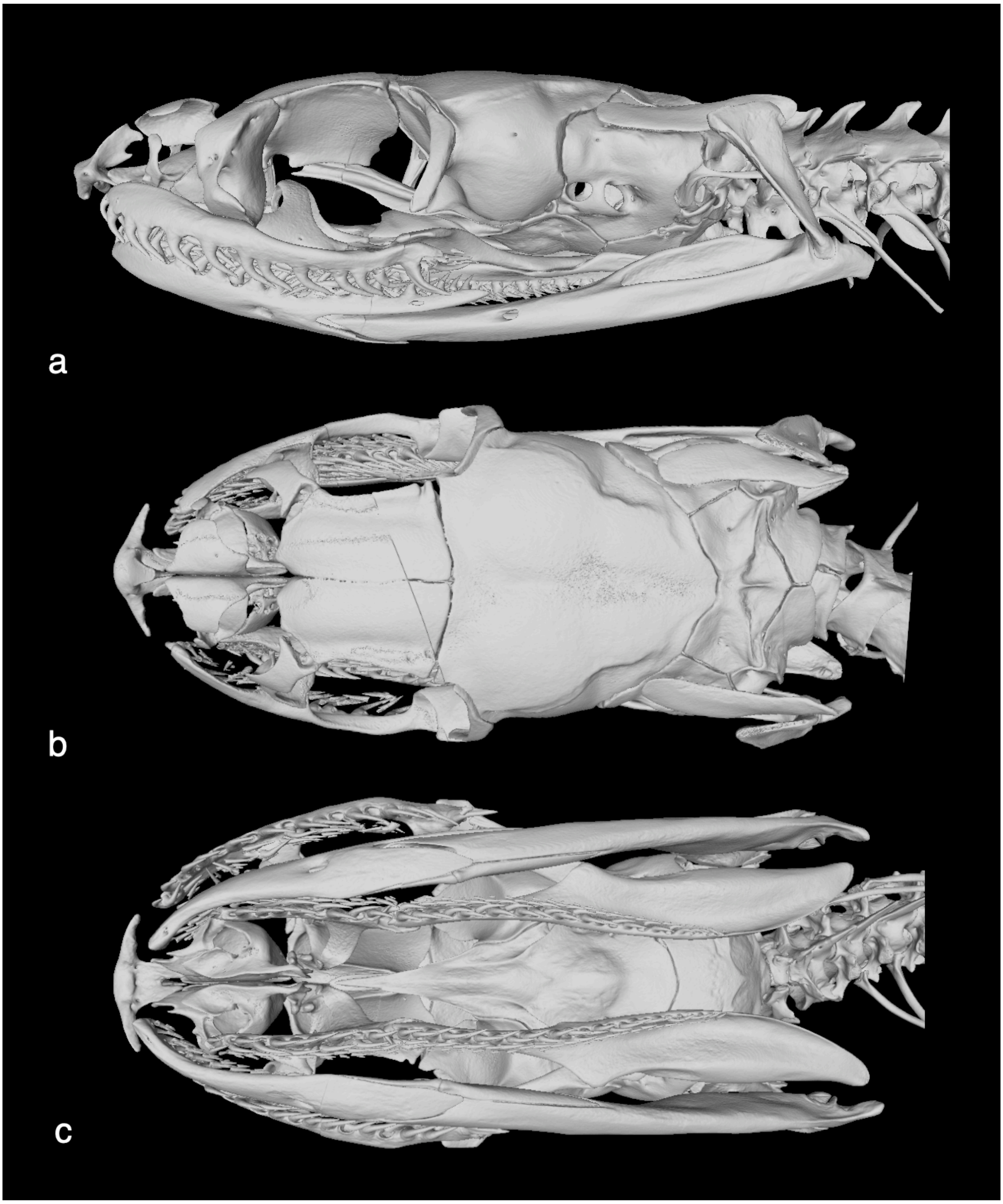

Fig. S28: Lateral (a), dorsal (b) and ventral (c) views of the cranium of *Buhoma procterae* (BE-RBINS-VER-REP-18541).

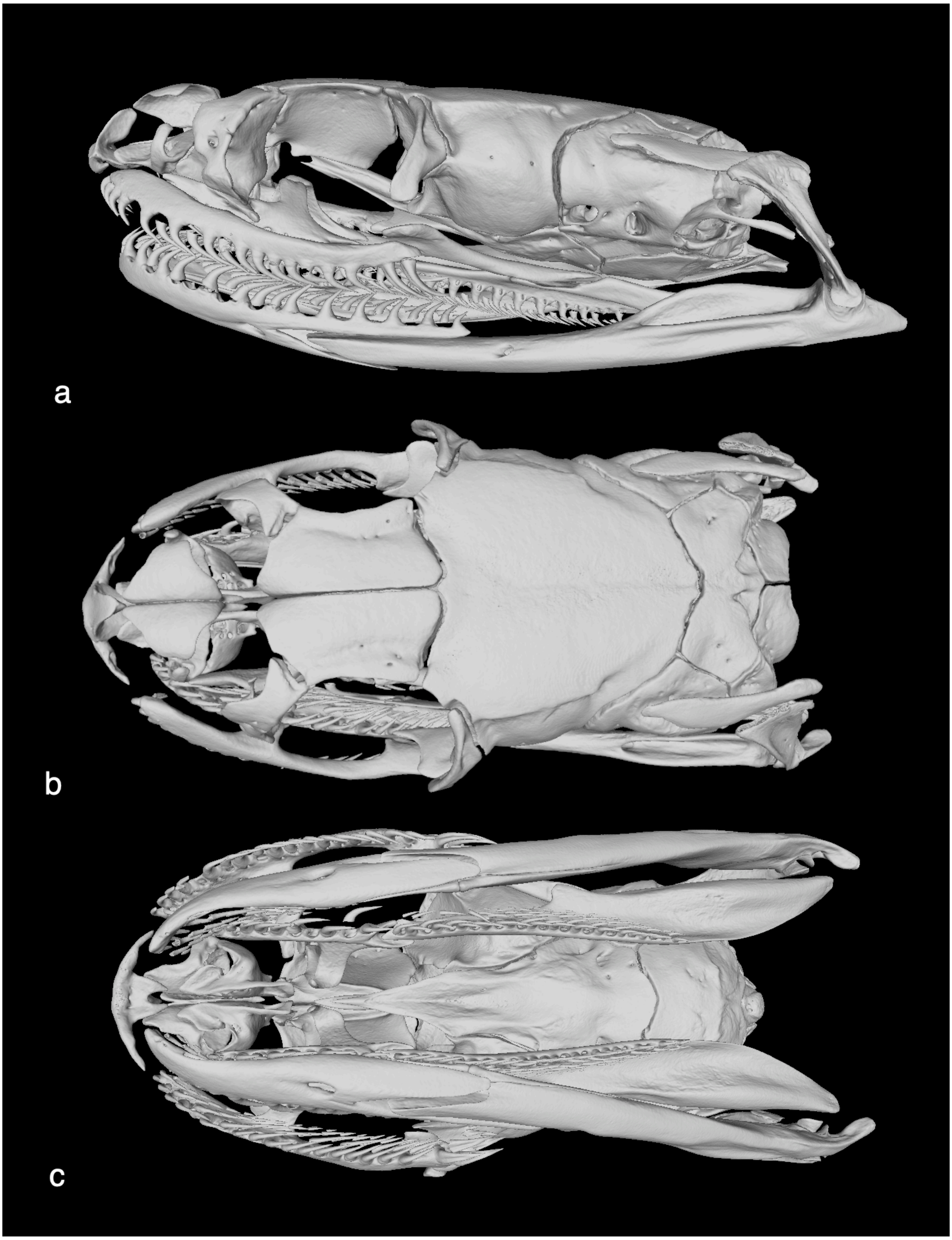

Fig. S29: Lateral (a), dorsal (b) and ventral (c) views of the cranium of *Buhoma vauerocegae* (BE-RBINS-VER-REP-18542).

Supplementary tables

Table S1 (next page): Accession number of traditional nuclear and mitochondrial loci. The accession numbers in blue are the sequences generated in this study; 'Reference genome' means the sequence of the traditional nuclear marker has been harvested from the reference genome; 'mitochondrion' means the sequence of the mitochondrial marker has been harvested from a complete mitochondrial genome; 'Alignment issue' means sequence for the particular locus is available for the species in question but it does not align with most of the other sequences that were used; the first column lists the names of the species in the UCE dataset (e.g., *Heteroliodon lava* contributes the UCE sequences whereas some of the traditional marker sequences are of *H. occipitalis*; in such cases, however, an overwhelming majority of sequence data is contributed by the former); the last column lists the names used for composites in the – traditional nuclear marker dataset (traditional mito-nuclear marker dataset) [traditional mitochondrial marker datasets]; composite names are formed from the first letter of the specific epithet (e.g., *Daboia* PSR = *Daboia palaestinae* + *siamensis* + *russelii*).

Family/subfamily	Species (named as in the UCE data)	*12s*	*16s*	*ND4*	*CYTB*	*BDNF*	*RAG1*	*RAG2*	*C-MOS*	Renaming of composites
Atractaspidinae	*Atractaspis reticulata*	NA	MK621470	MK621528	MK621586	NA	MK621501	NA	MK621643	
	Atractaspis irregularis	NA	MK621473	MK621570	MK621629	JN614212	MK621517	NA	MK621648	
	Aparallactus modestus	FJ404130	MG746954	MG775975	AY612007	EU402626 (*A. werneri*)	MG775761	FJ404405	MG775861	*Aparallactus MW*
	Homoroselaps lacteus	KX694590	MK621499	MK621583	MK621641	JQ599029	MK621524	FJ404411	MK621677	
	Polemon collaris	FJ404139	MG746931	MG775943	AY612029	NA	MG775736	FJ404414	MG775829	
	Polemon fulvicollis	NA	MG746942	MG775949	MG746837	NA	NA	NA	MG775837	
	Polemon graueri	NA	MG746939	MG775948	MG746835	NA	MG775742	NA	MG775836	
	Polemon robustus	NA	MG746937	MG775946	MG746831	NA	MG775739	NA	MG775832	
	Xenocalamus bicolor	FJ404141 (*X. transvaalensis*)	MG746906	MG775913	MG746796	NA	MG775716	NA	AY611934 (*X. transvaalensis*)	*Xenocalamus BT*
Buhoma	*Buhoma procterae*	FJ404148	AY611818	DQ486328	DQ486353	NA	NA	FJ404423	DQ486177	
	Buhoma depressiceps	FJ404147	OR295386	OR296691	OR296690	OR296686	OR296692	FJ404422	OR296688	
	Buhoma marlieri	NA	OR295387	Discarded	Discarded	OR296687	OR296693	NA	OR296689	
Cyclocoridae	*Cyclocorus lineatus*	NA	NA	NA	MG458749	NA	NA	NA	MG458759	
	Oxyrhabdium leporinum	NA	NA	NA	MN166286	NA	NA	FJ404466	MN166283	
	Oxyrhabdium modestum	NA	NA	NA	MG458756	NA	NA	NA	NA	
Psammodynastidae new family	*Psammodynastes pulverulentus*	MK065545	MK194116	KX660600 (*P. pictus*)	AF471031	Reference genome	Reference genome	Reference genome	Reference genome	*Psammodynastes PP (Psammodynastes pulverulentus)*
Elapidae	*Emydocephalus ijimae*	NC_066234 (mitochondrion)	NC_066234 (mitochondrion)	NC_066234 (mitochondrion)	NC_066234 (mitochondrion)	Reference genome	Reference genome	Reference genome	Reference genome	
	Elapsoidea boulengeri	AF544745 (*E. semiannulata*)	JF357946 (*E. semiannulatus*)	JF357928 (*E. semiannulata*)	AF039260 (*E. semiannulata*)	FJ433987 (*E. semiannulata*)	Alignment issue	EF144098 (*E. semiannulata*)	AF544678 (*E. semiannulata*)	*Elapsoidea semiannulata*
	Bungarus multicinctus	MK065561	MK194133	MN165167	AJ565002	Reference genome	Reference genome	Reference genome	Reference genome	
	Laticauda laticaudata	KX239655	KX239663	FJ606538	AB701328	Reference genome	Reference genome	Reference genome	Reference genome	
	Laticauda colubrina	KX239654	MK193892	EU546998	KX239643	Reference genome	Reference genome	Reference genome	Reference genome	
	Laticauda saintgironsi	NA	NA	FJ606507	NA	NA	NA	NA	NA	
	Hydrophis cyanocinctus	KX239648	MK194271	FJ593215	KX239640	Reference genome	Reference genome	Reference genome	Reference genome	
	Hydrophis curtus	KX239653	KJ653939	KJ653856	KJ653904	Reference genome	Reference genome	Reference genome	Reference genome	
	Hydrophis melanocephalus	MK775532 (mitochondrion)	MK775532 (mitochondrion)	MK775532 (mitochondrion)	MK775532 (mitochondrion)	Reference genome	Reference genome	Reference genome	Reference genome	
	Notechis scutatus	U96802	KU323981	EU547034 (*N. ater*)	LC640464	Reference genome	Reference genome	Reference genome	Reference genome	*Notechis SA (Notechis scutatus)*
	Naja naja	EU547088	GQ359756	MT346908	MT346712	Reference genome	Reference genome	Reference genome	Reference genome	
	Naja melanoleuca	U96801	MF497427	MH337394	MH337588	NA	MT347390	NA	AY611904	
	Ophiophagus hannah	JN687930	JN687931	MZ343823	MZ343887	Reference genome	Reference genome	Reference genome	Reference genome	
	Suta gouldii	NA	NA	MT151759	NA	NA	EU546889 (*Suta spectabilis*)	NA	EU546928 (*Suta spectabilis*)	*Suta GS (Suta spectabilis)*
	Walterinnesia aegyptia	U96807	HQ267785	MT346955	MT346782	NA	MT347441	NA	MT347033	
Lamprophiinae	*Boaedon fuliginosus*	FJ404165	AY611881	KM519696	KM519716	EU402646	KM519732	FJ404438	FJ404266	
	Boaedon olivaceus	FJ404170	KX277253	KM519697	KM519717	NA	KM519731	FJ404443	FJ404271	
	Boaedon perisilvestris	NA	KX249794	NA	NA	NA	NA	NA	NA	
	Boaedon radfordi	NA	NA	KM519694	KM519713	NA	KM519728	NA	KM519748	
	Bothrophthalmus lineatus	FJ404146	FJ404198	FJ404349	LC640409	NA	NA	FJ404421	FJ404251	
	Hormonotus modestus	FJ404159	FJ404195	FJ404360	FJ404296	NA	NA	FJ404433	FJ404261	
	Gracililima nyassae	FJ404182	AY611896	HQ207150	MF804432	NA	NA	NA	MF804462	

	Limaformosa chanleri	NA	NA	NA	MF804439	NA	NA	NA	MF804469	
	Lycopodion capense	FJ404178	AY611838	FJ404376	MT433521	EU402652	Alignment issue	FJ404450	AY611930	
	Mehelya poensis	FJ404183	AY611863	FJ404380	AY612045	NA	NA	FJ404454	AY611954	
Micrelapidae	*Micrelaps muelleri*	NA	NA	MG776013	MW075289	NA	NA	NA	MW075300	
Prosymninae	*Prosymna frontalis*	KR814630	KR814655	KR814707	MT482414	NA	MT460640	NA	MT460582	
	Prosymna sundevalli	NA	NA	NA	MT482424	NA	MT460650	NA	MT460590	
	Prosymna visseri	FJ404192	AY188072	NA	MT482427	NA	MT460652	FJ404463	FJ404292	
Psammophiinae	*Dipsina multimaculata*	NA	NA	DQ486332	DQ486357	NA	NA	NA	DQ486181	
	Hemirhagerrhis viperina	FJ404134 (*H. hildebrandtii*)	FJ404214 (*H. hildebrandtii*)	DQ486289	DQ486453	NA	NA	FJ404409 (*H. hildebrandtii*)	FJ404239 (*H. hildebrandtii*)	*Hemirhagerrhis HV (Hemirhagerrhis hildebrandtii)*
	Malpolon insignitus	DQ451951	AY611851	FJ404320 (*M. monspessulanus*)	AY612033	OL547595 (*M. monspessulanus*)	KY762208 (*M. monspessulanus*)	FJ404390 (*M. monspessulanus*)	AY611942	*Malpolon IM*
	Rhamphiophis rostratus	FJ404126	AY611897	FJ404329	DQ486394	JQ599049 (*R. oxyrhynchus*)	NA	FJ404401	DQ486187	*Rhamphiophis RO*
	Psammophis phillipsii	FJ404120	MK005716	MK032669	LC640454	NA	NA	FJ404395	AY611970	
	Psammophis schokari	HQ658448	HQ658453	MG003080	MG003028	NA	NA	FJ404396	MG002958	
Pseudaspidinae	*Pseudaspis cana*	FJ404187	AY611898	FJ404384	AY612080	NA	NA	FJ404458	AY058942	
	Pythonodipsas carinata	FJ404189	AY188075	FJ404386	AY188036	NA	NA	FJ404460	AY187997	
Pseudoxyrhophiinae	*Duberria shirana*	FJ404154 (*D. lutrix*)	KX694640 (*D. lutrix*)	LR798686 (*D. lutrix*)	LR798605	NA	KX695083 (*D. lutrix*)	FJ404428 (*D. lutrix*)	AF471138 (*D. lutrix*)	*Duberria SL (Duberria lutrix)*
	Heteroliodon lava	FJ404158 (*H. occipitalis*)	FJ404211 (*H. occipitalis*)	NA	FJ404308 (*H. occipitalis*)	NA	NA	FJ404432 (*H. occipitalis*)	FJ387211 (*H. occipitalis*)	*Heteroliodon occipitalis*
	Langaha madagascariensis	FJ404172	AY188059	FJ404370	AY188020	NA	NA	FJ404445	AY187981	
	Leioheterodon madagascariensis	KX694599	AF544797	FJ404371	KX694881	FJ433992	Alignment issue	EF144103	KX694824	
	Madagascarophis colubrinus	FJ404181 (*M. meridionalis*)	MG189475	MCU49313	AY586253	JQ073077 (*M. meridionalis*)	NA	KU925301	AY586226	*Madagascarophis CM*
Colubridae (outgroup)	*Dolichophis jugularis*	AY039152	AY376769	AY487046	AY486917	NA	NA	NA	AY486941	
	Spalerosophis diadema	AY039148	MG700257	AY487059	AY486926	NA	Alignment issue	NA	AY486950	
Homalopsidae (outgroup)	*Myanophis thanlyinensis*	NC_060375 (mitochondrion)	NC_060375 (mitochondrion)	NC_060375 (mitochondrion)	NC_060375 (mitochondrion)	Reference genome	Reference genome	Reference genome	Reference genome	
Viperidae (outgroup)	*Daboia palaestinae*	JN870183	AJ275775	DQ305477 (*D. russelii*)	MF140586	LC519947 (*D. siamensis*)	Reference genome	MK194871 (*D. siamensis*)	Reference genome	*Daboia PSR (Daboia siamensis) [Daboia PR]*

Table S2–S5: Accession number of query sequences used to perform BLAST search against a reference genome to locate and extract a particular nuclear gene.

Table S2

Query	Subject genome	Gene targeted
EU402646	*Psammodynastes pulverulentus*	*BDNF*
FJ433986	*Myanophis thalyinensis*	*BDNF*
FJ433990	*Hydrophis curtus*	*BDNF*
FJ433990	*Hydrophis melanocephalus*	*BDNF*
FJ433990	*Hydrophis cyanocinctus*	*BDNF*
FJ433990	*Emydocephalus ijimae*	*BDNF*
FJ433990	*Laticauda colubrina*	*BDNF*
FJ433990	*Laticauda laticaudata*	*BDNF*
EU402656	*Notechis scutatus*	*BDNF*
LC519961	*Ophiophagus hannah*	*BDNF*
FJ433990	*Bungarus multicinctus*	*BDNF*
LC533897	*Naja naja*	*BDNF*

Table S3

Query	Subject genome	Gene targeted
MG458761	*Psammodynastes pulverulentus*	*C-MOS*
JX463013	*Myanophis thanlyinensis*	*C-MOS*
KT965865	*Hydrophis curtus*	*C-MOS*
KT965865	*Hydrophis melanocephalus*	*C-MOS*
KT965865	*Hydrophis cyanocinctus*	*C-MOS*

KT965865	*Emydocephalus ijimae*	*C-MOS*
AF544702	*Laticauda colubrina*	*C-MOS*
AF544702	*Laticauda laticaudata*	*C-MOS*
EU546944	*Notechis scutatus*	*C-MOS*
KX694798	*Ophiophagus hannah*	*C-MOS*
AY058924	*Bungarus multicinctus*	*C-MOS*
EU366445	*Naja naja*	*C-MOS*
AF471156	*Daboia siamensis*	*C-MOS*

Table S4

Query	Subject genome	Gene targeted
MT347390	*Psammodynastes pulverulentus*	*RAG1*
EU546908	*Myanophis thanlyinensis*	*RAG1*
EU546889	*Hydrophis curtus*	*RAG1*
EU546889	*Hydrophis melanocephalus*	*RAG1*
EU546889	*Hydrophis cyanocinctus*	*RAG1*
EU546908	*Emydocephalus ijimae*	*RAG1*
EU546908	*Laticauda colubrina*	*RAG1*
EU546908	*Laticauda laticaudata*	*RAG1*
EU546905	*Notechis scutatus*	*RAG1*
MZ753612	*Ophiophagus hannah*	*RAG1*
JF357954	*Bungarus multicinctus*	*RAG1*
MT347390	*Naja naja*	*RAG1*
EU546908	*Daboia siamensis*	*RAG1*

Table S5

Query	Subject genome	Gene targeted
MK194960	*Psammodynastes pulverulentus*	*RAG2*
EF144097	*Myanophis thanlyinensis*	*RAG2*
MK195117	*Hydrophis curtus*	*RAG2*
MK195117	*Hydrophis melanocephalus*	*RAG2*
MK195117	*Hydrophis cyanocinctus*	*RAG2*
MK195117	*Emydocephalus ijimae*	*RAG2*
MK194731	*Laticauda colubrina*	*RAG2*
MK194731	*Laticauda laticaudata*	*RAG2*
MK195117	*Notechis scutatus*	*RAG2*
MK194875	*Ophiophagus hannah*	*RAG2*
MK195078	*Bungarus multicinctus*	*RAG2*
MK195061	*Naja naja*	*RAG2*

Table S6: MorphoSource ark number+links to the slices and 3D volumes of the micro-CT scans generated in this study

**Catalogue number**	**Species**	**Slices**	**3DModel**
BE-RBINS-VER-REP-43	*Ophiophagus hannah*	http://n2t.net/ark:/87602/m4/541884	http://n2t.net/ark:/87602/m4/541004
BE-RBINS-VER-REP-19300	*Naja melanoleuca*	http://n2t.net/ark:/87602/m4/541887	http://n2t.net/ark:/87602/m4/541011
BE-RBINS-VER-REP-19104	*Naja annulata*	http://n2t.net/ark:/87602/m4/541890	http://n2t.net/ark:/87602/m4/541017

BE-RBINS-VER-TYPES-2612	*Elapsoidea trapei*	http://n2t.net/ark:/87602/m4/541893	http://n2t.net/ark:/87602/m4/541034
BE-RBINS-VER-REP-8408	*Pseudaspis cana*	http://n2t.net/ark:/87602/m4/541039	http://n2t.net/ark:/87602/m4/541896
BE-RBINS-VER-REP-19125	*Mehelya poensis*	http://n2t.net/ark:/87602/m4/541899	http://n2t.net/ark:/87602/m4/541043
BE-RMCA-VER-R.30641	*Mehelya nyassae*	http://n2t.net/ark:/87602/m4/541072	http://n2t.net/ark:/87602/m4/541082
BE-RBINS-VER-REP-18885	*Boaedon olivaceus*	http://n2t.net/ark:/87602/m4/541052	http://n2t.net/ark:/87602/m4/541054
BE-RMCA-VER-REP-14864	*Lycodonomorphus subtaeniatus*	http://n2t.net/ark:/87602/m4/541669	http://n2t.net/ark:/87602/m4/541671
BE-RBINS-VER-REP-18542	*Buhoma vauerocegae*	http://n2t.net/ark:/87602/m4/541059	http://n2t.net/ark:/87602/m4/541061
BE-RMCA-VER-REP 18091	*Buhoma marlieri* Holotype	http://n2t.net/ark:/87602/m4/543336	http://n2t.net/ark:/87602/m4/543342
BE-RBINS-VER-REP-8620	*Buhoma marlieri*	http://n2t.net/ark:/87602/m4/541067	http://n2t.net/ark:/87602/m4/541075
BE-RBINS-VER-REP-16404	*Buhoma depressiceps*	http://n2t.net/ark:/87602/m4/541080	http://n2t.net/ark:/87602/m4/541091
BE-RBINS-VER-REP-18541	*Buhoma procterae*	http://n2t.net/ark:/87602/m4/541676	http://n2t.net/ark:/87602/m4/541678
RBINS 9284	*Psammophis mossambicus*	http://n2t.net/ark:/87602/m4/541683	http://n2t.net/ark:/87602/m4/541688
BE-RBINS-VER-REP-561	*Malpolon monspessulanus monspessulanus*	http://n2t.net/ark:/87602/m4/541692	http://n2t.net/ark:/87602/m4/541694
BE-RBINS-VER-REP-6719	*Lycodonomorphus inornatus*	http://n2t.net/ark:/87602/m4/541699	http://n2t.net/ark:/87602/m4/541906

BE-RBINS-VER-REP-5069	*Rhamphiophis rostratus*	http://n2t.net/ark:/87602/m4/541703	http://n2t.net/ark:/87602/m4/541908
BE-RBINS-VER-REP-18633	*Kladirostratus acutus*	http://n2t.net/ark:/87602/m4/541712	http://n2t.net/ark:/87602/m4/541910
BE-RMCA-VER-R.11	*Prosymna ambigua bocagei* Holotype	http://n2t.net/ark:/87602/m4/541707	http://n2t.net/ark:/87602/m4/541914
BE-RBINS-VER-REP-11733	*Prosymna sundevalli*	http://n2t.net/ark:/87602/m4/541881	http://n2t.net/ark:/87602/m4/541912
